# Theoretical Elucidation of β-O-4 Bond Cleavage of Lignin Model Compound Promoted by Sulfonic Acid-Functionalized Ionic Liquid

**DOI:** 10.3389/fchem.2019.00078

**Published:** 2019-02-15

**Authors:** Yaqin Zhang, Feng Huo, Yanlei Wang, Yu Xia, Xin Tan, Suojiang Zhang, Hongyan He

**Affiliations:** Beijing Key Laboratory of Ionic Liquids Clean Process, CAS Key Laboratory of Green Process and Engineering, State Key Laboratory of Multiphase Complex Systems, Institute of Process Engineering, Chinese Academy of Sciences, Beijing, China

**Keywords:** lignin, ionic liquid, DFT, molecular dynamics, β-O-4 bond, reaction mechanism

## Abstract

While the depolymerization of lignin to chemicals catalyzed by ionic liquids has attracted significant attention, the relevant molecular mechanism, especially the cleavage of specific bonds related to efficient depolymerization, still needs to be deeply understood for the complexity of this natural aromatic polymer. This work presents a detailed understanding of the cleavage of the most abundant β-O-4 bond in the model system, guaiacylglycerol β-guaiacyl ether, by a Brønsted acidic IL (1-methyl-3-(propyl-3-sulfonate) imidazolium bisulfate ([C_3_SO_3_Hmim][HSO_4_]) using density functional theory calculation and molecular dynamics simulation. It has been found that [C_3_SO_3_Hmim][HSO_4_] generates zwitterion/H_2_SO_4_ *via* proton transfer with an energy barrier of 0.38 kcal/mol, which plays a dominant role in the lignin depolymerization process. Subsequently, the reaction can be carried out *via* three potential pathways, including (1) the dehydration of α-C-OH, (2) dehydration of γ-C-OH, and (3) the protonation of β-O. The electrophilic attack of H_2_SO_4_ and the hydrogen-bonding interaction between GG and zwitterion are the two most important factors to promote the depolymerization reaction. In all steps, the dehydration of α-C-OH route is computed to be favored for the experiment. The relatively higher energy barrier for β-O-4 bond dissociation among these reaction steps is attributed to the hindrance of the self-assembled clusters of GG in the mixed system. Further, the dense distribution of H13([C_3_SO_3_Hmim]) surrounding O21(GG), indicated by sharp peaks in RDFs, reveals that -SO_3_H in cations plays a substantial role in solvating lignin. Hopefully, this work will demonstrate new insights into lignin depolymerization by functionalized ILs in biomass conversion chemistry.

## Introduction

Lignin, a main aromatic component of lignocellulosic biomass accounting for 18–40 wt% of dry wood (Amen-Chen et al., 2001; Zhang et al., 2019), is an alternative feedstock to the depleting petroleum-based sources owing to its abundant reserves, renewability and low cost (Zakzeski et al., 2010). The basic building blocks of lignin are coniferyl, sinapyl, and *p*-coumaryl alcohols which are mostly linked by β-O-4, 4-O-5, α-O-4, β-5, β-1, and 5-5 bonds to form a three-dimensional amorphous and irregular structure. The dominate bond between monomers is the β-O-4 ether bond, representing ~50% of all linkages (Chatel and Rogers, 2014), and the main linkage is the key objective and the major challenge of most utilization studies (Jia et al., 2010a,b; Younker et al., 2011; Lu et al., 2016). Other bonds are becoming increasingly difficult to degrade during traditional processing methods because of strong carbon-carbon bond formation (Upton and Kasko, 2016). Owing to its complex structure, lignin is resistant to degradation, thus, it is generally burned to produce electricity after the bioethanol production (Zakzeski et al., 2010). Therefore, preparation of fine aromatic chemicals from lignin residues by target cleavage of β-O-4 bond is a promising strategy. Recently, efforts have been devoted to the decomposition of β-O-4 ether bond by solvolysis (Ma et al., 2014; Deepa and Dhepe, 2015; Katahira et al., 2016), oxidation (Prado et al., 2016), reduction (Shuai et al., 2016), pyrolysis (Jollet et al., 2014) etc. However, these technologies need to conduct under extreme reaction conditions to achieve degradation of lignin, and these processes mostly involve strong acid, caustic alkali, volatile toxic solvents, or noble metals (Venica et al., 2008). In this aspect, greener utilization of lignin is urged.

Ionic liquids (ILs) have attracted enormous attention as an environmental benign medium for solubilizing lignocelluloses (Mora-Pale et al., 2011; Brandt et al., 2013; D'Anna et al., 2014), owing to their unusual properties such as near-zero vapor pressure, tunable structure of cations and anions, and excellent solvent power for both organic and inorganic substances (Zhang et al., 2006, 2017a), etc. The recent experiments have elucidated the catalytic depolymerization of real lignin in ILs to aromatic compounds, through breaking β-O-4 ether bonds. Jia et al. reported that the hydrolysis of β-O-4 bonds of lignin dimer [guaiacylglycerol-β-guaiacyl ether (GG)], could be achieved in an acidic IL (1-H-3-methylimidazolium chloride), and a possible reaction mechanism accounting for the primary product (guaiacol) was speculated (Jia et al., 2010a). After that, Cox et al. demonstrated that the coordination of anions of ILs with hydroxyl groups can stabilize the intermediates, which facilitate the cleavage of β-O-4 ether bond and inhibit the condensation of lignin fragments during fragmentation (Cox et al., 2011). Further studies have shown that the combination of [Bmim][Cl] with metal chloride was effective for β-O-4 bond cleavage, which is attributed to hydrochloric acid formed by hydrolysis of metal chlorides (Jia et al., 2010b). Recent studies have presented that the selective tailor for ester bonds, rather than the ether bonds of lignin, yields methyl p-hydroxycinnamate with [Bmim][FeCl_4_]. The high catalytic activity was attributed to the narrow HOMO-LUMO gap between ester lignin and [FeCl_4_]^−^ anion (Li et al., 2018). The efficient degradation of lignin using acidic IL, 1-(4- sulfobutyl)-3-methylimidazoliumhydrosulfate ([C_4_H_8_SO_3_Hmim][HSO_4_]), was investigated (Long et al., 2015), yielding useful fine aromatic chemicals such as guaiacol, phenol and 4-ethylphenol through dehydration and dealkylation of lignin. Amarasekara et al. reported that sulfuric acid functionalized IL exhibited better catalytic activity than pure sulfuric acid at the same H^+^ ion concentration because it acts as both a solvent and a catalyst (Amarasekara and Wiredu, 2011). Cai et al. developed an emulsion reactor containing butyl-3-(butyl-4-sulfonate) imidazolium hydrogen sulfate for lignin depolymerization, which exhibited high efficiency in product separation by automatic phase partition (Cai et al., 2015). Recently, Singh et al. compared the depolymerization of lignin in various ILs catalysts and sulfuric acid (Singh and Dhepe, 2016). Their results showed that [C_3_SO_3_Hmim][HSO_4_] had a superior capacity to synthesize low molecular-weight aromatic products with a high conversion rate, indicating that IL is a promising substitute for metals and inorganic catalysts to depolymerize real lignin. The enhanced catalytic activity of –SO_3_H functionalized ILs has resulted from the strong interaction between lignin moieties and ILs, which facilitates the hydrolysis of β-O-4 bonds (Janesko, 2014). Apart from the theoretical study of acid-promoted cleavage of β-O-4 bond (Qu et al., 2015), there are still some challenges to overcome as the reaction mechanism of ILs catalyzed depolymerization of lignin remains unclear. The acting mechanism of –SO_3_H functionalized ILs has not been elucidated yet, and this encourages us to perform a DFT mechanistic study. Our previous work explained the geometric and energetic details between various ILs and the lignin model compound, GG. It was found that ILs with sulfonic acid group had the strongest interaction with GG *via* hydrogen bonding and π-π interaction. Furthermore, hydroxyl groups and ether oxygen of GG are the key sites for the interaction (Zhang et al., 2017b). To further elucidate the reaction mechanism and physicochemical phenomena of β-O-4 bond cleavage with sulfonic IL, the conversion of GG to guaiacol by a –SO_3_H functionalized IL [C_3_SO_3_Hmim][HSO_4_] was proposed in this article by density functional theory (DFT) calculations and molecular dynamics (MD) simulations. The Gibbs energies of reaction profiles in vacuum and solvent, electron density at bond critical points, bond dissociation energies and non-covalent interactions were specially investigated to study the nature of β-O-4 bond cleavage. The computed results are expected to be helpful for understanding the mechanism of –SO_3_H functionalized IL-catalyzed transformation of lignin to small phenols.

## Theoretical Methods

### Quantum Chemistry Calculations

In this study, all DFT calculations were performed by employing Gaussian 09 software package (Frisch et al., 2013). The stationary points including reactants, intermediates, products, and transition states were optimized using the M06-2x functional, which is proposed to be excellent for describing the dispersion effects within noncovalent interactions (Zhao and Truhlar, 2008), combined with the standard 6–31+g(d,p) basis set (Hariharan and Pople, 1973; Frisch et al., 1984). Harmonic frequency calculations were carried out at the same level of theory to verify the optimized structures as zero imaginary frequency for minima and one imaginary frequency for transition states. The intrinsic reaction coordinate (IRC) calculations were traced to confirm the reaction pathways of transition states (Fukui, 1981). Structures at the two ends of IRC paths were optimized to minima, which represent the stable geometrics of reactants and products (A rigid scan of intermediates can be found in Figure S7 and the energies in Table S3). Single point energies and Gibbs free energies of all structures were refined at the M06-2x/6-311+g(d,p) level. Additionally, to mimic the solvent effect on the reactions, the solvation model based on density (SMD) in methanol solvent was adapted for all gas phase structures (Marenich et al., 2009; Bernales et al., 2012).

### Molecular Dynamics Simulations

MD simulations for GG-IL systems containing 400 GG and 430 pairs of [C_3_SO_3_Hmim][HSO_4_] were performed with the Gromacs 5.1.1 software package. The number of GG and ion pairs were based on the dissolution experiment of Singh et al. (Singh and Dhepe, 2016). The CHARMM general force field was used for cations [C_3_SO_3_Hmim]^+^ and anions [HSO_4_]^−^ (Jo et al., 2008; Vanommeslaeghe et al., 2010; Moyer et al., 2018), and the CHARMM parameters reported by Petridis and Smith (2009) were employed for lignin dimer GG. The starting structures of all the GG-IL systems were randomly built by PACKMOL (Martinez and Martinez, 2003). For the GG-IL mixtures, periodic boundary conditions (PBC) were used in x, y, z directions with an initial box size 90 × 90 × 90 Å, and the PME algorithm were used for electrostatic interactions. The systems were initially energy-minimized for 10,000 steps using the conjugate gradient algorithm to remove abnormal contacts between molecules. Subsequently, the systems were simulated for 5 ns under the NVT canonical ensemble with a V-rescale thermostat to increase the initial temperature to 500 K. Then, another 5 ns annealing were taken by decreasing the temperature from 500 to 300, 420, 440, 460, and 480 K under the NPT isothermal-isobaric ensemble with a V-rescale thermostat to reach equilibrium, respectively (Berendsen et al., 1984). Afterwards, the production runs were equilibrated for another 30 ns under canonical ensemble to collect the data of interest. The simulation results were obtained by analyzing the last 10 ns trajectory of production runs. For all MD simulations, the Lennard-Jones interaction was truncated at a radius of 1.2 nm, and the Coulombic interaction was treated using particle-mesh Ewald (PME) summation with a cutoff of 1.2 nm (Zheng et al., 2018).

## Results and Discussion

### Generation of Zwitterion/H_2_SO_4_ Complex

**Figure 1:**
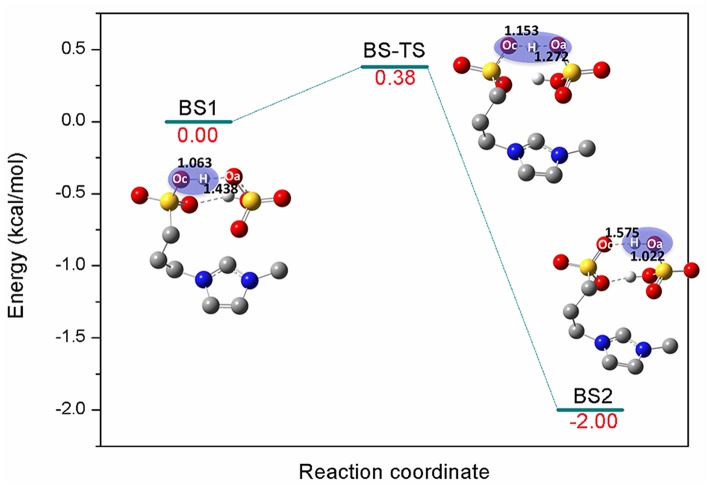
Potential energy profile for the generation of zwitterion/H_2_SO_4_ complex. The unimportant hydrogen atoms are omitted for clarity and the bond lengths are labeled in Å.

[C_3_SO_3_Hmim][HSO_4_] is one kind of sulfonic acid functionalized ILs (SAFILs). The class of Brønsted acidic ILs has displayed good characteristics for use as liquid catalysts in organic synthesis and for the hydrolysis of carbohydrates (Cole et al., 2002; Kitaoka et al., 2004; Qiao et al., 2004; Zare et al., 2012). It has also been reported that intermolecular hydrogen bonds were automatically formed between the anion and cation by the O-H…O hydrogen bonds (Liu et al., 2009), and the extremely strong hydrogen bonds resulted in proton transfer from cation to anion. Finally, the complex of zwitterion/H_2_SO_4_ was formed (Ohno et al., 2018). In the present work, the reaction barrier forming zwitterion/H_2_SO_4_ was investigated; H_2_SO_4_ was supposed to serve as better proton shuttle and zwitterion was expected to stabilize the intermediates or transition states. Here, a one-step reaction was identified in Figure 1. The precursor of the reaction **BS1** is a pair of [C_3_SO_3_Hmim][HSO_4_] bonded by O-H…O hydrogen bonds. The bond length of Oc-H (Oc, oxygen atom of cation) is 1.063 Å and the distance between Oa (Oa, oxygen atom of anion) and H (H, hydrogen atom of -SO_3_H) is 1.438 Å. Due to the strong nucleophilic ability of the anion, [HSO_4_]^−^ extracts the proton from [C_3_SO_3_Hmim]^+^ *via* Oc-H…Oa. In the transition state **BS-TS**, Oc-H bond is elongated to 1.153 Å and H…Oa gets closer to 1.272 Å. It is observed that the zwitterion/H_2_SO_4_ complex is formed by deprotonation of [C_3_SO_3_Hmim]^+^ in **BS2**. One can see that the covalent bond Oa-H is 1.022 Å and the distance between Oc and H is 1.575 Å. In this step, the calculated energy barrier is only 0.38 kcal/mol, and the product is 2 kcal/mol more stable than the reactant, suggesting a slightly exothermic reaction for this route. The existence of zwitterion/H_2_SO_4_ is considered to be geometrically and thermodynamically feasible at ambient temperature (Sun et al., 2014). In the subsequent mechanism study, the zwitterion/H_2_SO_4_ complex was used as the main catalyst for the bond cleavage reactions. In the following sections, detailed mechanistic calculations of route A, B, and C were shown to understand the depolymerization of lignin with this zwitterion/H_2_SO_4_.

### Route A: Dehydration of α-C-OH

**Figure 2:**
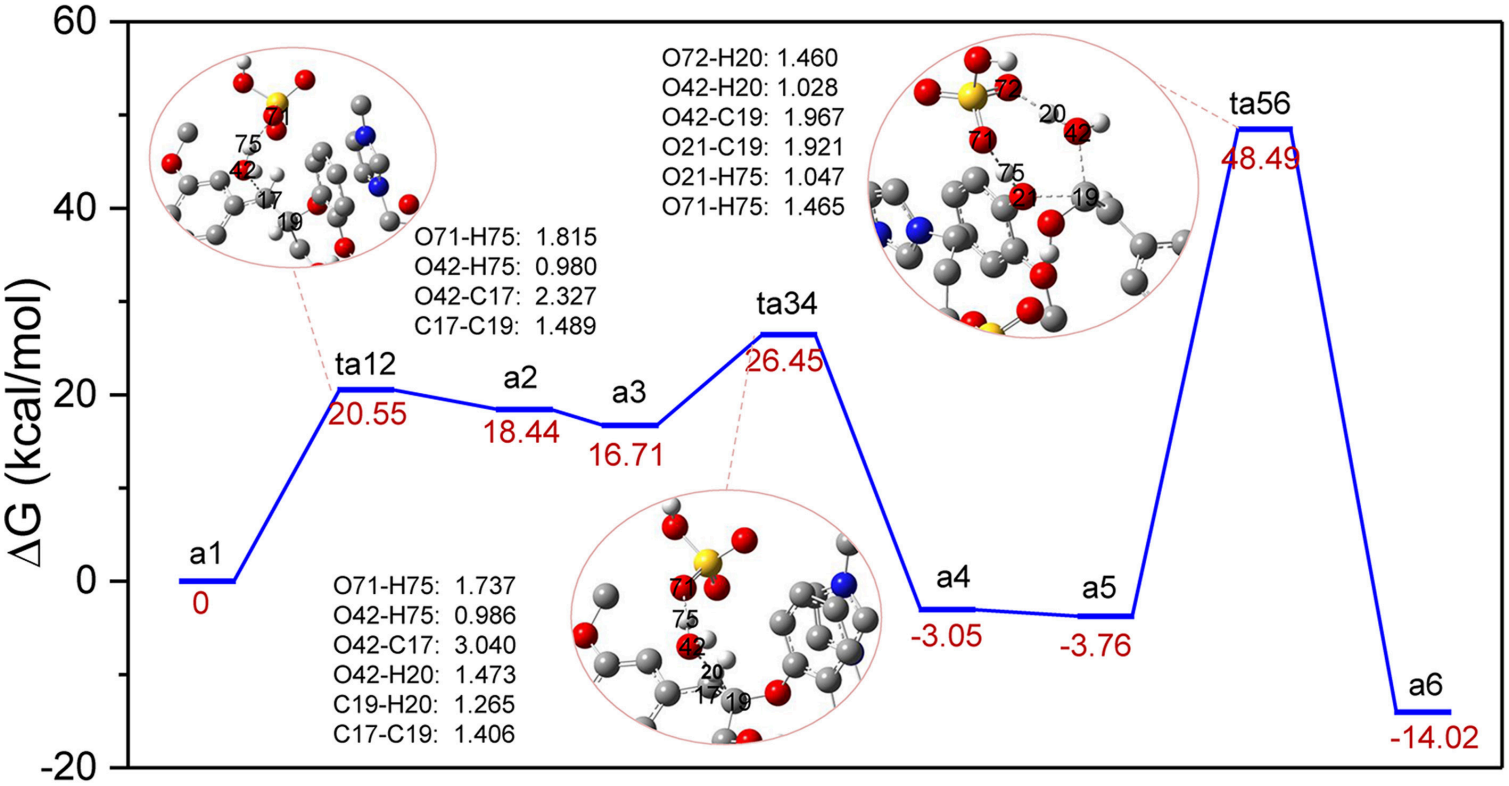
Gibbs free energy profile for β-O-4 bond cleavage undergoing α-C-OH dehydration. The unimportant hydrogen atoms are omitted for clarity and the bond lengths are labeled in Å.

The previous experimental studies have speculated that the depolymerization of lignin to phenols by acids or acidic ionic liquids experienced a dehydration process (Cox et al., 2011; Janesko, 2014), which is presented in route A. It is believed that route A involves dehydration of α-C-OH, followed by hydrolysis of the β-O-4 ether bond by the water molecules that were removed (Scheme S1 in ESI). For the system studied with zwitterion/H_2_SO_4_ acting as a catalyst, the reaction profile of Gibbs free energy was shown in Figure 2. The bond cleavage process was conducted *via* three elementary steps: the elimination of α-C-OH, followed by the deprotonation of β-C and finally the hydrolysis of the β-O-4 bond. Figure 2 displays the computed energy profile, where the zero-energy reference point, **a1**, is taken as the initial complex between a GG molecule and the zwitterion/H_2_SO_4_. The first transition state, **ts12**, represents the elimination of α-C-OH, where H_2_SO_4_ acts as a proton shuttle, donating a proton H75 at O71 of H_2_SO_4_. At this stage the distance between O71 and H75 is elongated to 1.815 Å. The proton H75 approaches O42 and protonation of O42 also leads to elongation of O42-C17, resulting in the removal of the hydroxyl group at C17 and the formation of an intermediate, **a2**. The distances of O42-H75, O42-C17, and C17-C19 are 0.980, 2.327, and 1.489 Å, respectively. The first protonation step involves an energy barrier of 20.55 kcal/mol. Before the subsequent deprotonation reaction, **a2** evolves into a more stable conformation (**a3)** *via* the rearrangement of GG and zwitterion. After that, **a3** is converted to **a4** through **ts34**, realizing the removal of H20 at C19 and the recovery of H_2_SO_4_ by another proton shuttle, H_3_O^+^. Noted, the H20 removed gets close to the eliminated H_2_O molecule to form H_3_O^+^, then H_3_O^+^ returns H75 to [HSO_4_]^−^ and the dehydration step is complete. The energy barrier of **a3** to **a4** is 9.74 kcal/mol, which is considerably lower than that of the first protonation step (20.55 kcal/mol). Next, **a4** evolves into a more energetically stable structure, **a5**, which is geometrically feasible to conduct the bond cleavage step, **ta56**. In this step, the proton H75 of H_2_SO_4_ attacks O21 of β-O-4 bond, and the bond length of O71-H75 is elongated to 1.465 Å. Meanwhile, the adjacent water molecule approaches β-C, C19. Due to the formation of hydrogen bonds between O72 and H20, the covalent bond O42-H20 is weakened, causing H20 to transfer from O42 to O72, and the remaining part of the water molecule is added to C19. The final step 3 is to form the O21-H75, C19-O42 bonds and to break O21-C19 bond to yield the final phenolic products. The energy barrier of **a5** to **a6** is 52.25 kcal/mol, which is the highest among all steps in route A (all structures can be found in Figure S1). The possible reason for this high energy barrier is the steric hindrance that occurred in the β-O-4 site, which will be further discussed in the MD simulation section. Additionally, due to the flexibility of the lignin molecule (Upton and Kasko, 2016), π-π stacking interaction between imidazolium ring of zwitterion and benzene ring of lignin GG is considered to be the key factor which stabilizes the transition states and intermediates.

### Route B: Dehydration of γ-C-OH

**Figure 3:**
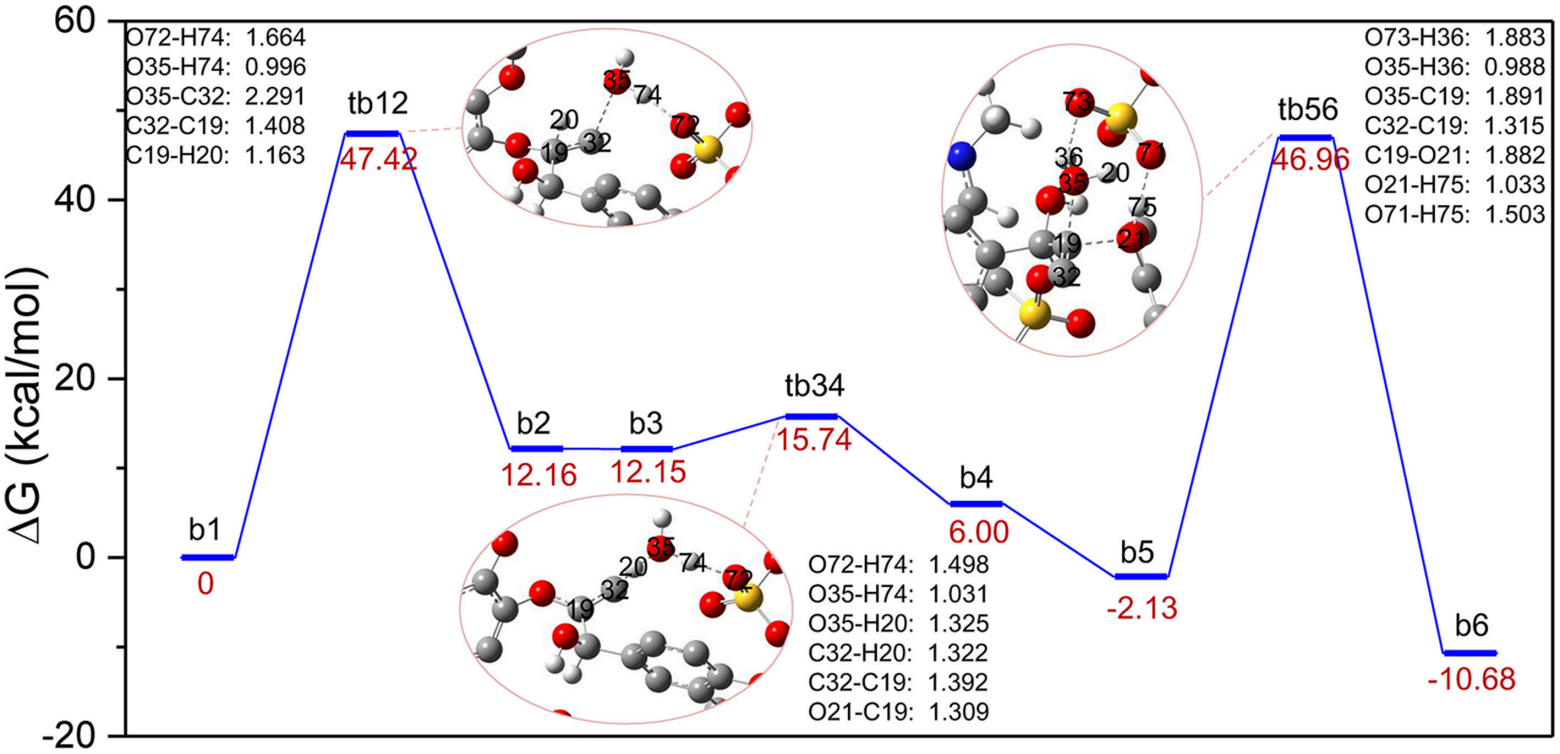
Gibbs free energy profile for β-O-4 bond cleavage undergoing γ-C-OH dehydration. The unimportant hydrogen atoms are omitted for clarity and the bond lengths are labeled in Å.

The major reaction steps of route B are identified in Figure 3 and all geometric structures along this route can be found in Figure S2. Similarly, the bond cleavage is also carried out *via* three elementary steps: the elimination of γ-C-OH accompanied by intramolecular hydrogen rearrangement, followed by the deprotonation of γ-C, and finally the hydrolysis of the β-O-4 bond. The reaction steps start from the deprotonation of H_2_SO_4_. Once H_2_SO_4_ is deprotonated, the resulting H74 proton attacks O35 *via* the γ-C-OH site. The bond length of O72-H74 is elongated to 1.664 Å, and bond H74-O35 has the distance of 0.996 Å. At the same time, bond C32-O35 is weakened to 2.291 Å. As a result, the hydroxyl group O35-H and H74 leave the GG molecule in the form of one water molecule. In this step, conversion of **b1** to **b2** experiences the transition state, in the form of **tb12**, by overcoming an energy barrier of 47.42 kcal/mol, which is much higher than that of the protonation of α-C-OH in route A. One reason is that γ-C-OH tends to form intramolecular hydrogen bonds with the adjacent methoxy group, which make it difficult to break (Zhang et al., 2017b), and the other is probably owing to the rearrangement of hydrogen H20 from C19 to C32 in **tb12**. One can see that C32 is bonded with two hydrogen atoms after the removal of the hydroxyl group O35-H. The strong electronegativity of C32 attracted H20 of C19, which makes intermediate **b2** stable. Upon the transfer of H20 from C19 to C32, H20 forms H_3_O^+^ with the water molecule generated in the first step, and these changes suggest that C19-C32 is migrating from a C-C single bond to a C = C double bond in the dehydration intermediate **b2** (same with **b3**). The transition state **tb34** clearly reveals that H_3_O^+^ returns H74 to O72, and H20 leaves with O35-H in the form of a water molecule. At the same time, the bond length of C19 = C32 in **tb12** (1.392 Å) is shorter than that in **tb34** (1.408 Å). The departing of H20 overcomes an energy barrier of 3.59 kcal/mol. Afterwards, intermediate **b4** evolves into a more stable conformational isomer, **b5**, which is geometrically feasible to conduct the cleavage of the β-O-4 bond. Subsequently, **b5** is converted to the phenolic product **b6** through **tb56**, where the hydrogen atom H75 is abstracted by O21 of lignin; co-occurring with the proton H35 transfer from H_2_O to [HSO_4_]^−^ and the addition of O35-H20 to C19. The energy barrier of this step is 49.09 kcal/mol. Comparing with that of **tb34**, the length of ether bond C19-O21 in **tb56** is elongated from 1.309 to 1.882 Å. Figure 3 demonstrates that the overall process is exothermic by 10.68 kcal/mol, and the first protonation step is the rate-determining step. However, the bond cleavage step remains to be a high energy barrier due to the steric hindrance at the β-O-4 site.

### Route C: Protonation of β-O

**Figure 4:**
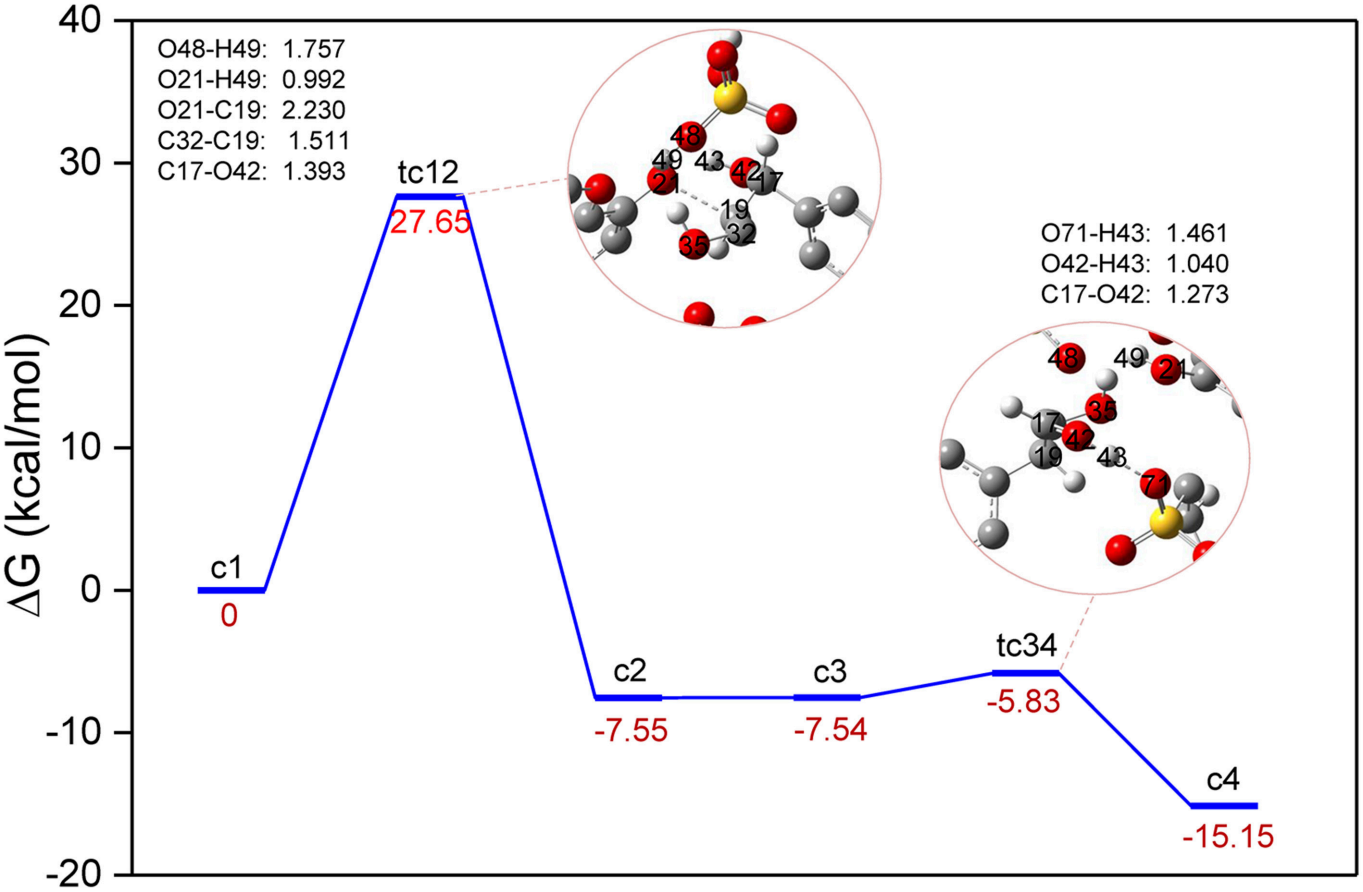
Gibbs free energy profile for β-O-4 bond cleavage undergoing directly β-C-O protonation. The unimportant hydrogen atoms are omitted for clarity and the bond lengths are labeled in Å.

The conversion processes in Route A: Dehydration of α-C-OH and Route B: Dehydration of γ-C-OH have identified the three steps *via* protonation, elimination, and hydrolysis, and the hydrolysis process was generally speculated by the experimental work (Jia et al., 2010a; Cox et al., 2011; Singh and Dhepe, 2016). As reported by Loerbroks et al. (2013), cellobiose can be activated by acid hydrolysis of glycosidic linkage of cellobiose, with a low energy barrier of 33 kcal/mol. Here, we reported the mechanism of direct protonation of β-O-4 ether bond by zwitterion/H_2_SO_4_. A two-step reaction mechanism is shown in Figure 4, and the structures of route C can be found in Figure S3. Starting from protonated lignin GG, **c1**, the first step involves the dissociation of the O21-C19 bond, followed by a conformational change of the position between C17, C19, and C32. Due to the elongation of O21-C19, C19 gets too close to the carbon atom of the benzene ring which is originally bonded with C17, so that C19 replaces the position of C17. Consequently, C19 in structure **c2** becomes bonded to the carbon atom of the benzene ring, and both C17 and C32 become connected with C19. The computed energy barrier to form **tc12** is 27.98 kcal/mol. followed by the intermediate **c2**, at which point the hydroxyl group, O42-H43 at C17, gets close to the SO_3_ part of zwitterion. Accordingly, the hydrogen atom H43 transfers from O42 to O71 and the ion pair [C_3_SO_3_Hmim][HSO_4_] is re-obtained. The reaction barrier of the second step is estimated to be 1.71 kcal/mol. It is worth pointing out that there is experimental evidence showing that the activation of the O-glycosidic site could be hindered by the preferential protonation of O of hydroxyl group in cellulose (Palkovits et al., 2010; Rinaldi et al., 2010). Though route C experiences the lowest energy barrier, a catalyst is likely to be hindered by the hydroxyl groups, so the dehydration processes displayed in route A and B are more likely to be initiated. These findings may be concerted to the mechanism speculated by Singh et al. (Singh and Dhepe, 2016). The relatively higher energy barriers in dehydration processes could be attributed to much larger bond dissociation energies of O-H bonds than those of β-C-O ether bond, which were verified to be 112.39 and 117.25 kcal/mol for (α-C-O)-H and γ-C-O-H, respectively, and 74.46 kcal/mol for the β-C-O bond (Figure S4 and Table S2 in ESI).

### Solvent Effect

**Figure 5:**
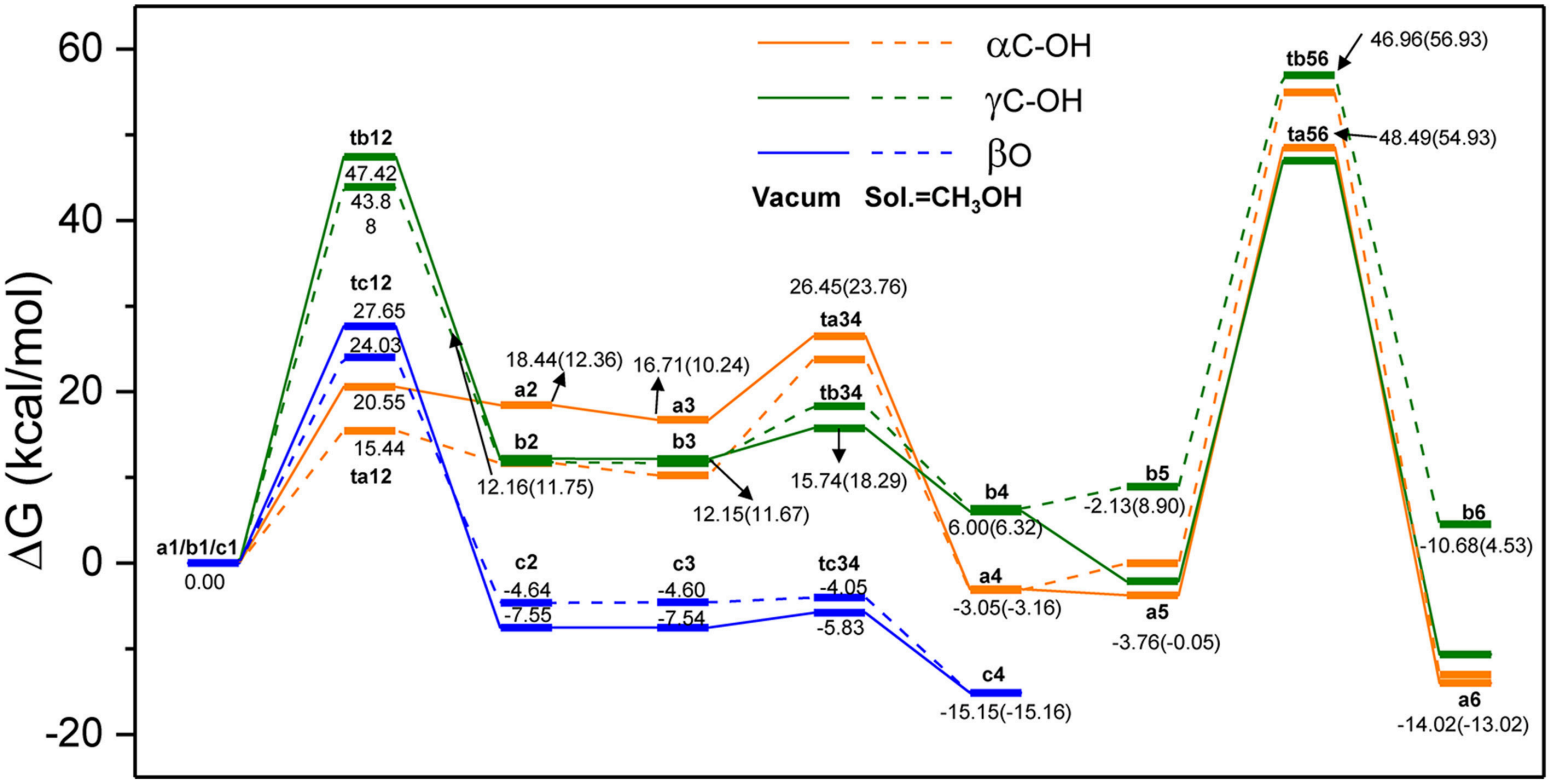
The overall reaction pathways of route A: α-C-OH, route B: γ-C-OH, and route C: β-O with solid lines representing energies in vacuum condition and dash lines representing energies in solvent (SMD, sol. = CH_3_OH).

In order to evaluate the solvation effect on the reaction profiles, the Gibbs free energies were refined at the M06-2x/6-311+g(d,p) level of theory. According to experimental results, methanol was chosen as the SMD solvent because of its fine performance for lignin depolymerization (Singh and Dhepe, 2016; Li et al., 2018). The energy profiles of the three reaction routes are shown in Figure 5; the energies under vacuum are denoted in solid lines and those under solvent condition are denoted in dash lines. Overall, the energies for the species of **ta12**, **ta34**, **tb12**, and **tc12** under solvent condition are lower than that under vacuum condition, which suggests that the solvent effects strongly stabilize these transition states. However, the energies for the species of **ta56**, **tb34**, **tb56**, and **tc34** are relatively higher than that under vacuum condition. The different degrees of stabilization of intermediates and transition states causes the reaction kinetics to be significantly affected. In route A (α-C-OH), the relative activation energies of **ta12**, **ta34**, and **ta56** are reduced by 5.11 kcal/mol and increased by 3.78 and 2.73 kcal/mol, respectively. In route B (γ-C-OH), the energy barriers of **tb12** and **tb56** are lowered by 3.54 and 1.06 kcal/mol, respectively. The energy barrier of **tb34** rises by 3.03 kcal/mol. In route C, the energy barrier of **tc12** and **tc34** is reduced by 3.62 and 1.16 kcal/mol, respectively. The detailed activation energies of all the transition states are given in Table 1. Through comparing the energy profiles of possible pathways of β-O-4 bond cleavage, it is clear that the most kinetically favored pathway could be route A, with the lowest activation energy of 15.44 kcal/mol. This is concerted to the calculation results in the gas phase. Furthermore, analysis of solvent effects on the β-O-4 bond cleavage reaction indicates that methanol solution has a better stabilization effect on the protonation of the hydroxyl group at α-C and γ-C, and the protonation of β-O in the initial steps. However, the solvent effects have a lower stabilization effect on the transition states in the hydrolysis steps and give little help to the bond cleavage, which may be attributed to the competition between methanol molecules and water molecules to form hydrogen bonds with H_2_SO_4_, especially in **ta56**. Therefore, water molecules have some difficulty in approaching the β-O-4 ether bond. Among the two hydrolysis routes, the energy barrier of route A is generally lower than route B, which proves that α-C-OH is a better reaction site (Zhang et al., 2017b), among the two hydrolysis routes.

**Table 1:** Activation energies of transition states in Figure 5.

**Route A (α-C-OH)**	**ta12**	**ta34**	**ta56**
Energy barrier (kcal/mol)	20.55 (15.44)	9.74 (13.52)	52.25 (54.98)
Route B (γ-C-OH)	tb12	tb34	tb56
Energy barrier (kcal/mol)	47.42 (43.88)	3.59 (6.62)	49.09 (48.03)
Route C (β-O)	tc12	tc34	
Energy barrier (kcal/mol)	27.65 (24.03)	1.71 (0.55)	

*Energies under solvent condition are displayed in parentheses*.

### Non-covalent Interactions Analysis

**Figure 6:**
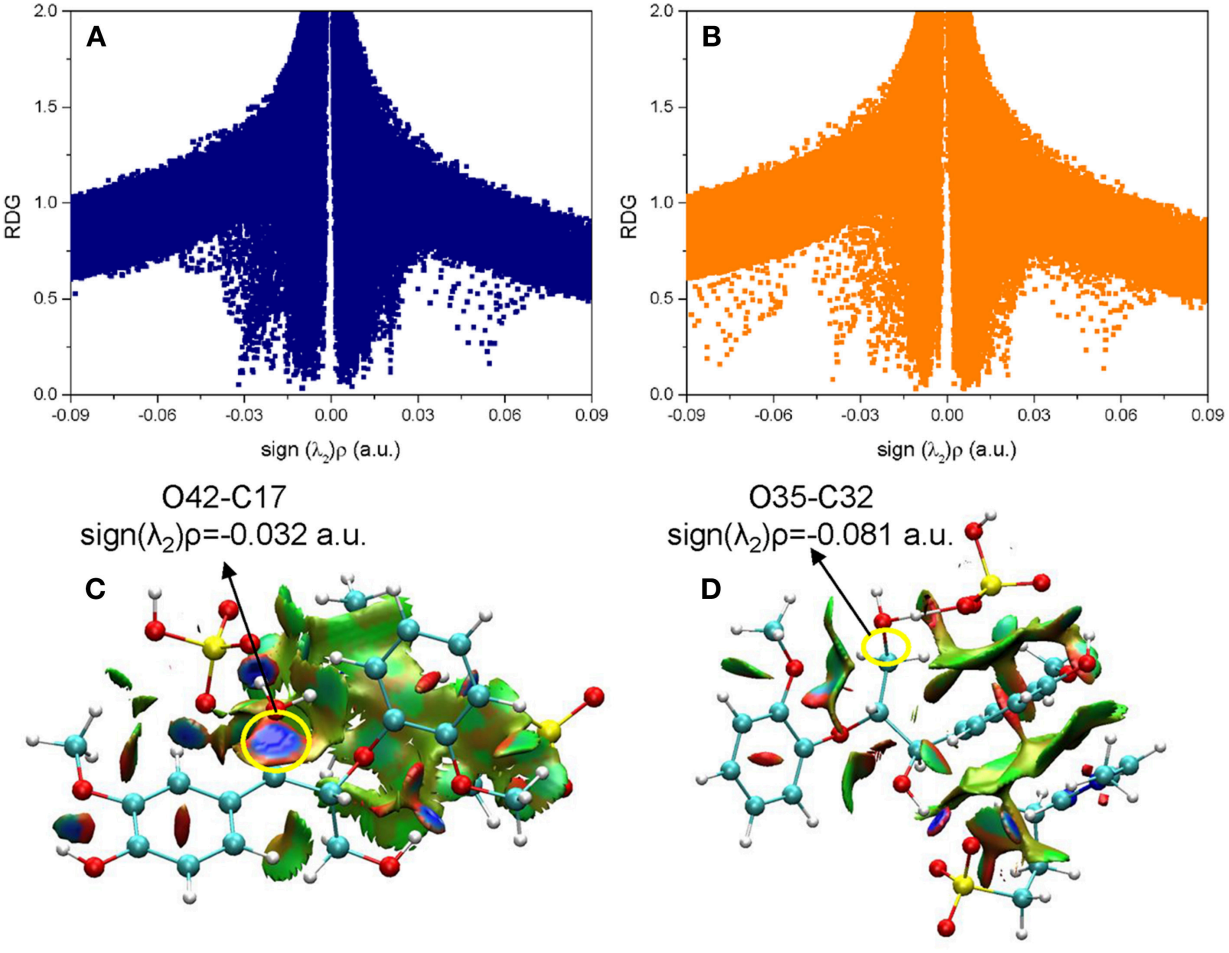
The noncovalent interactions in the protonation steps in **(A)** ta12 and **(B)** tb12, and the corresponding 3D plots **(C,D)** are shown below with blue regions indicating strong electrostatic interactions, red regions indicating steric hindrance and green regions indicating more dispersive attractive interactions. The 3D plots are given with an isosurface of 0.7 a.u..

**Figure 7:**
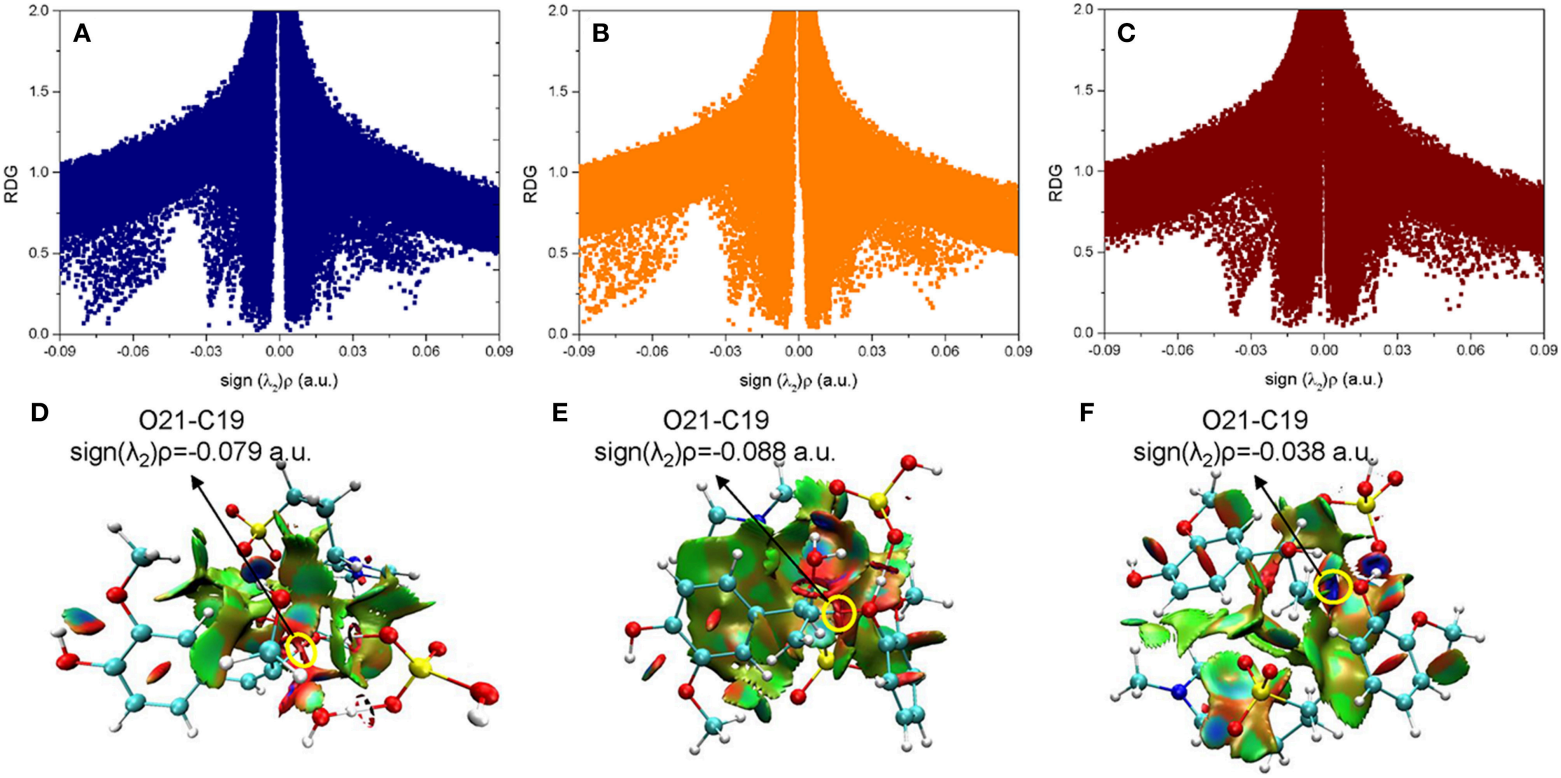
The noncovalent interactions in the β-O-4 bond breaking steps in **(A)** ta56, **(B)** tb56, and **(C)** tc12, and the corresponding 3D plots **(D–F)** are shown below with blue regions indicating strong electrostatic interactions, red regions indicating steric hindrance, and green regions indicating more dispersive attractive interactions. The 3D plots are given with an isovalue of 0.7 a.u..

Besides the reaction pathways, the non-covalent interactions between lignin GG and catalyst are investigated for the most important steps according to the atoms in molecules (AIM) theory (Cioslowski, 1991; Bader, 2002; Johnson et al., 2010). The inter- and intramolecular weak interactions are differentiated by the sign of second Hessian eigenvalue [sign(λ_2_)ρ] in Reduced density gradient (RDG) analysis, and visualized by 3D plots with a color-scale from −0.03 to 0.02 a.u. The description of noncovalent interactions can be found within these work (Yang et al., 2016; Zhang et al., 2017b). The electronic properties are shown for transition states of protonation steps (**ta12** and **tb12**) in Figure 6 and bond cleavage steps (**ta56**, **tb56**, and **tc12**) in Figure 7. For the protonation steps, electron densities at the bond critical point of O42-C17 (α-C and O of hydroxyl group at α-C) and O35-C32 (γ-C and O of hydroxyl group at γ-C) are focused on the following analysis, relating to the departure of the hydroxyl group. While for the bond cleavage steps, electron densities at bond critical point of O21-C19 (β-C-O) are investigated with the scatter plots and their corresponding 3D plots. As can be seen, the electron density at O42-C17 is 0.032 a.u. with a positive Laplacian value of 0.092 a.u. (Table S1 in ESI), and the blue surface denoted by yellow circle (Figure 6C) indicates that O42 still maintains a strong electrostatic-dominated noncovalent interaction with C17. The weak interaction also proves that protonation of the hydroxyl group at C17 strongly weakened the O42-C17 bond. Similarly, the electron density at O35-C32 is 0.081 a.u. with a negative Laplacian value of −0.142 a.u. The negative Laplacian value usually means that the electron density is locally concentrated, which is dominated by a covalent interaction. The site denoted by a yellow circle (Figure 6D) does not indicate that a noncovalent interaction surface is consistent with the presence of the O35-C32 covalent bond. Moreover, the lower electron density at O42-C17 than O35-C32 suggests that the hydroxyl group at α-C is more easily activated by H_2_SO_4_. Meanwhile, other large green surfaces are classified by π-π stacking interactions between benzene rings of lignin GG and the imidazolium ring of zwitterion.

In a quest to better understand the difference between the dehydration route and protonation route, the electron density properties at the bond critical point of O21-C19 (β-C-O ether bond), of transition states (**ta56**, **tb56**, and **tc12**) in the bond cleavage steps, are shown in Figure 7. One can see that, there are obvious spikes at where sign(λ_2_)ρ equals to −0.079, −0.088, and −0.038 a.u. in Figures 7A–C, respectively. There is a dense distribution of spikes at a position where sign(λ_2_)ρ approximately equals to zero, which indicates that the van der Waals dominated dispersive attractions between zwitterion and GG molecules. These interactions are shown by green surfaces in Figures 7D–F. However, the regions which correspond to the O21-C19 bond interaction for **ta56** and **tb56** are in red color, and that for **tc12** are in blue color. The bigger electron densities at BCP of O21-C19 of **ta56** and **tb56** suggest that O21 and C19 are strongly bonded, meaning there are obvious repulsive effects at the red regions. On the contrary, the electron density at BCP of O21-C19 of **tc12**, 0.038 a.u. is much smaller than the former, which demonstrates that the O21-C19 bond is greatly weakened in the protonation route. Additionally, the nonbonded atoms, O21 and C19 show a strong electrostatic attraction at the blue regions. Owing to the stronger attack of H_2_SO_4_ and dispersion effects of the zwitterion, route C has the lowest reaction barrier in the bond cleavage step.

### Radical Distribution Functions

**Figure 8:**
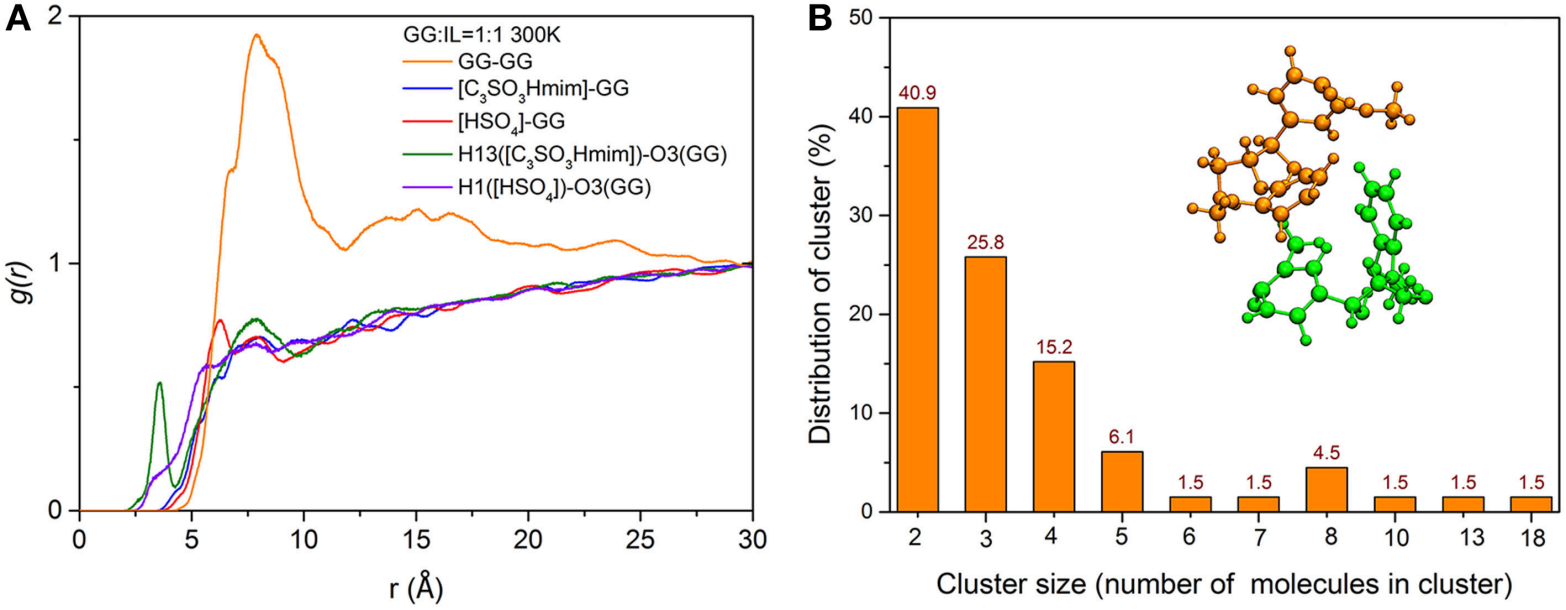
**(A)** RDFs between center of mass of GG and GG, [C_3_SO_3_Hmim]^+^ and GG, [HSO_4_]^−^ and GG, atom-atom RDFs between H13([C_3_SO_3_Hmim]) and O21(GG), H1([HSO_4_]) and O21(GG); **(B)** The number distribution of clusters with different numbers of GG molecules in the system of 300 K, and the snapshot of a typical cluster with two GG molecules.

To gain a deeper understanding of how the location of ILs and GG molecules affects the cleavage of the β-O-4 bond, the structural properties of cations and anions close to the ether-oxygen atom are quantified by radical distribution functions between center of mass of GG and GG (GG-GG), cation [C_3_SO_3_Hmim]^+^ and GG (C_3_SO_3_Hmim-GG), anion [HSO_4_]^−^ and GG ([HSO_4_]-GG), and atom-atom distribution between H13 of [C_3_SO_3_Hmim]^+^ and O3 of GG, H1 of [HSO_4_]^−^ and O3 of GG. Here, H13 is the hydrogen atom of -SO_3_H group in [C_3_SO_3_Hmim]^+^ and O21 is the ether-oxygen atom of GG. As shown in Figure 8A, the [C_3_SO_3_Hmim]-GG and [HSO_4_]-GG RDFs do not show obvious peaks, indicating the less structured GG-IL orientation. One shoulder at ~6 Å in Figure 8A suggests that there are more anions in the solvation layer than cations, and anions are closer to GG, which is in agreement with Moyer's results (Moyer et al., 2018). Though the RDFs of center of mass do not show a significant difference between [C_3_SO_3_Hmim]-GG and [HSO_4_]-GG, the atom-atom RDFs in Figure 8A do. As shown, H13 has a dense distribution around O21, with a sharp maximum at ~3.5 Å and a second maximum at ~7.5 Å. The first sharp peak implies the solvation shell which is ascribed to the hydrogen-bonding interaction between ether-oxygen O21 and H13 of -SO_3_H group. It is speculated that a mass of H-bonds are directly formed between O21 and H13, resulting in the very sharp peaks observed at ~3.5 Å in the RDF curves. Meanwhile, the second peak is farther away from O21, which is possibly due to the occupancy by the acidic hydrogens of the imidazolium ring. The close contacts between the imidazolium ring and ether-oxygen lead to -SO_3_H group locating at a far distance. In contrast to H13([C_3_SO_3_Hmim])-O21(GG) RDFs, the H1([HSO_4_])-O21(GG) RDFs show little variation to [HSO_4_]-GG, and only weak peaks are located at ~7 Å, which implies that the -SO_3_H group of cations are more closely located around GG. The unobvious RDF peaks between IL and GG are likely to be ascribed to strong self-aggregation of GG, as indicated by sharp peaks between GG and GG. The strong self-aggregation results in steric hindrance to direct protonation of β-O in route C. It was found that GG clusters are distributed with different numbers of GG molecules (Figure S6 and Figure 8) and the majority of the clusters include two GG molecules (Figure 8B). With increasing temperature, a gradual loss of local solvation layers is observed, shown by the decrease in the solvation peaks (Figure S5). However, the shape of the first peak does not change a lot, proving the important role of -SO_3_H in solvating lignin. Moreover, a significant rise in temperature is more conducive to the dispersion of lignin in ionic liquids. Although the RDFs confirm that H13 of cation [C_3_SO_3_Hmim]^+^ has more favorable distances to ether-oxygen, this phenomenon is not contradicted by the formation of zwitterions/H_2_SO_4_ between [C_3_SO_3_Hmim]^+^ and [HSO_4_]^−^, because the reaction energy barrier is only 0.38 kcal/mol. In this case, protons get into inner solvation shells of ILs.

## Conclusion

DFT calculations have shown the mechanism details of the conversion of lignin model compound, guaiacyl glycerol-β-guaiacyl ether (GG), to guaiacol by -SO_3_H functionalized IL. Three possible pathways exist that contribute to the cleavage of the β-O-4 bond. Route A is characterized by the dehydration of α-C-OH, route B describes the dehydration of γ-C-OH, and route C involves the direct protonation of β-O of GG to form guaiacol species. H_2_SO_4_/zwitterion complexes are formed by basic steps, H_2_SO_4_ acts as a proton donor like a proton shuttle, while the zwitterion part provides a polar environment to stabilize the intermediates and transition states. The closer distance between H13([C_3_SO_3_Hmim])-O21(GG) in RDFs also confirmed that - SO_3_H in cations plays a substantial role in solvation of lignin. The computed energy barriers of the three routes indicated that the cleavage of the β-O-4 bond is more easily carried out through route C. However, the protonation of β-O-4 ether bond in route C could be hindered due to the steric effect of zwitterion, as pointed out by the experiments. Therefore, the dehydration routes A and B are more consistent with experimental results. In addition, the solvation model calculations strongly suggest that methanol is beneficial for protonation steps in routes A and B, not only for protonation of the β-O-4 bond in route C. These theoretical findings provide a mechanistic understanding of the cleavage of the β-O-4 bond of lignin with –SO_3_H functionalized ILs.

## Author Contributions

HH and SZ designed the research. YZ, FH, and YW carried out the whole simulation. YZ, FH, YW, and HH did data analysis. YZ, YX, and XT discussed the results and did the revisions. YZ, HH, and SZ wrote the manuscript.

### Conflict of Interest Statement

The authors declare that the research was conducted in the absence of any commercial or financial relationships that could be construed as a potential conflict of interest.
