# Supplementary material for: Theoretical Elucidation of β-O-4 Bond Cleavage of Lignin Model Compound Promoted by Sulfonic Acid-Functionalized Ionic Liquid
Source: Front Chem. 2019 Feb 15;7:78. doi: 10.3389/fchem.2019.00078 (PMC6384239; doi:10.3389/fchem.2019.00078)
Supplement: Supplementary file 1 [file Table_1.DOCX]

Supporting information for:

**Theoretical elucidation of β-O-4 bond cleavage of lignin model compound promoted by sulfonic acid-functionalized ionic liquid**

Yaqin Zhang^†^, Feng Huo^†^, Yanlei Wang^†^, Yu Xia^†^, Xin Tan^†^, Suojiang Zhang^†^, Hongyan He^†,^ *

^†^ *Beijing Key Laboratory of Ionic Liquids Clean Process, Key Laboratory of Green Process and Engineering, State Key Laboratory of Multiphase Complex Systems, Institute of Process Engineering, Chinese Academy of Sciences, Beijing 100190, China*

*^*^ Corresponding author. E-mail:* [*hyhe@ipe.ac.cn*](mailto:hyhe@ipe.ac.cn)

Scheme S1: The possible reaction mechanism for cleavage of β-O-4 bond by IL [C_3_SO_3_Hmim][HSO_4_].

Figure S1: Geometrics of reactants, intermediates, transition states and products in route A. The unimportant hydrogen atoms in the structures are omitted.

Figure S2: Geometrics of reactants, intermediates, transition states and products in route B. The unimportant hydrogen atoms in the structures are omitted.

Figure S3: Geometrics of reactants, intermediates, transition states and products in route C. The unimportant hydrogen atoms in the structures are omitted.

Table S1: Electron properties at bond critical points of transition states.

Figure S4: Routes of bond dissociation of lignin model, GG. Bond dissociation energies are summarized in Table S2.

Table S2: Bond dissociation energy of different types of bonds in model GG at M06-2x/6-311+G(d,p) level. Bonds to break are indicated in blue color.

Figure S5: Atom-atom RDFs of the system GG and [C_3_SO_3_Hmim][HSO_4_] at different temperatures.

Figure S6: Snapshots of GG clusters in the system of 300K. Left: three GG molecules in a cluster, right: four GG molecules in a cluster. The different molecules are shown in different colors.

Table S3: Rigid scanning to perform the conformational search at the b3lyp/6-31g(d,p) level and the lowest-energy conformation was marked by red circles. The dihedral of the lowest-energy conformation was compared with the original dihedral of the structure which was optimized from IRC path at the m062x/6-31+g(d,p) level.

Figure S7: Stable geometries of the interaction between GG and zwitterion/H2SO4 which were optimized at the B3LYP/6-31g(d,p) level and interaction energies were refined at the M06-2X/6-311+g(d,p) level with counterpoise method.


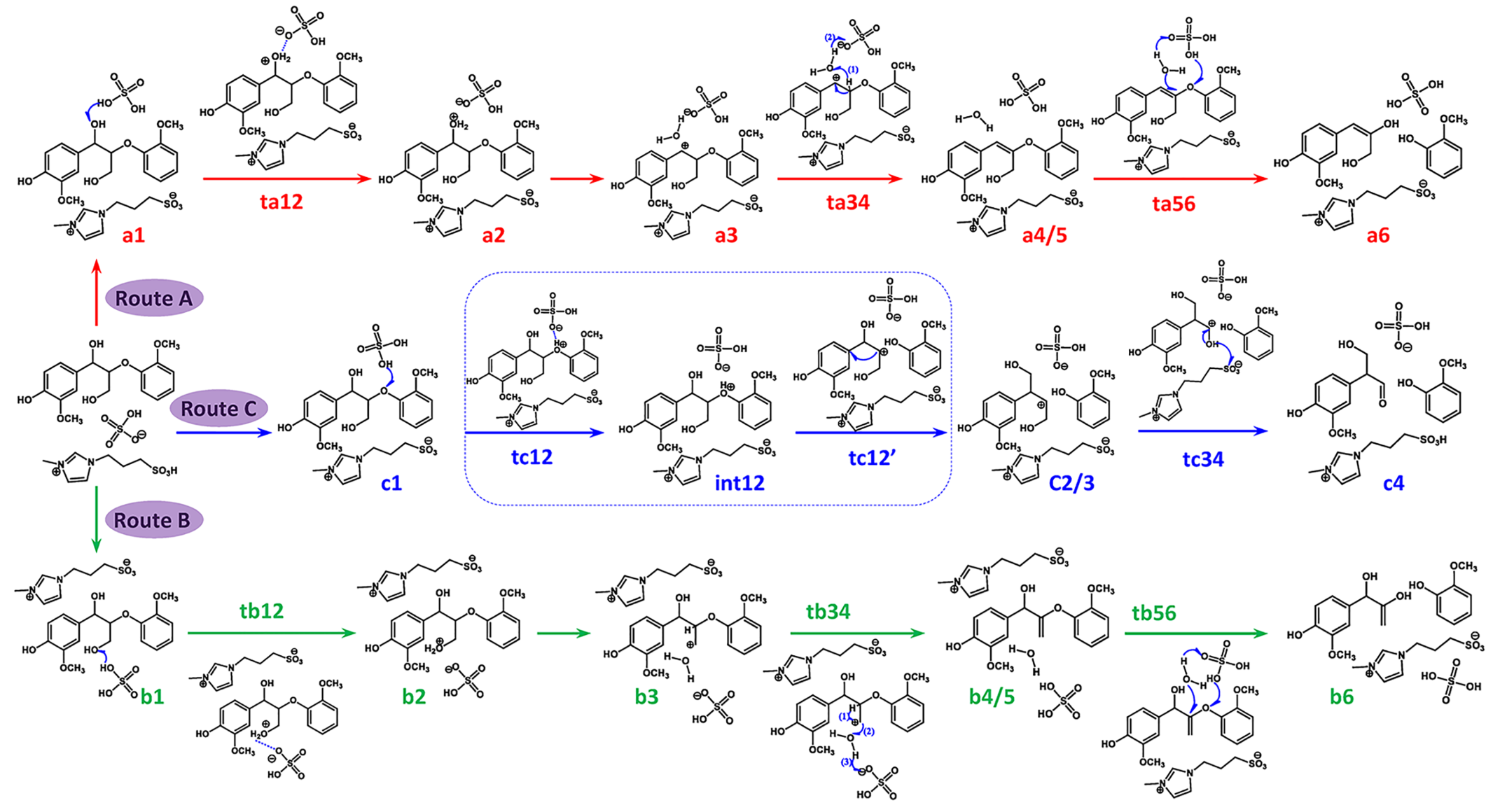


Scheme S1 The possible reaction mechanism for cleavage of β-O-4 bond by IL [C_3_SO_3_Hmim][HSO_4_].

| 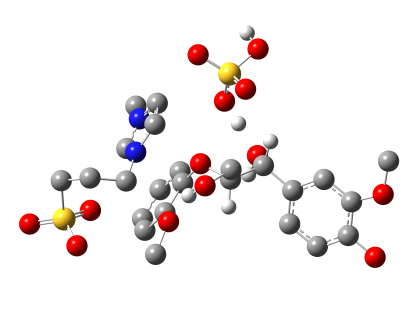 | 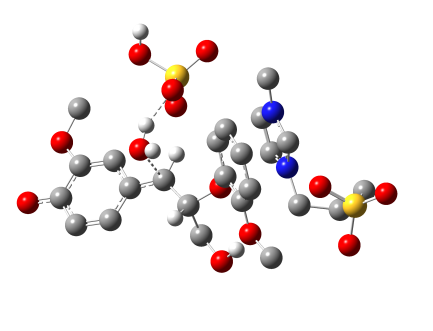 | 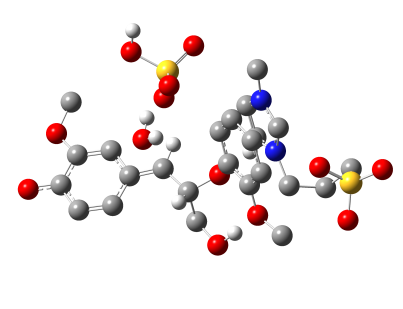 |
| --- | --- | --- |
| a1 | tsa12 | a2 |
| 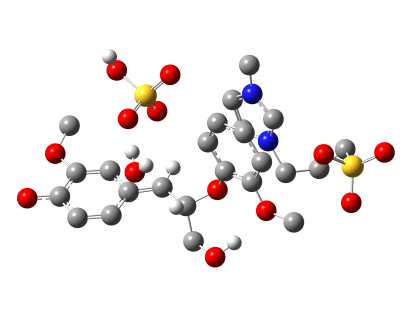 | 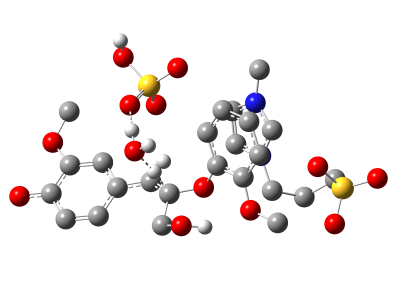 | 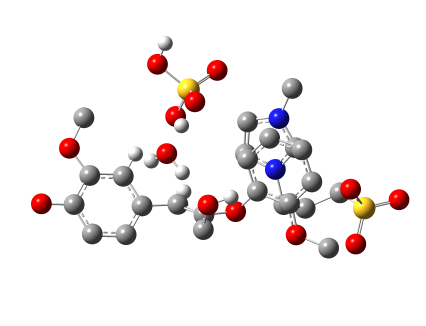 |
| a3 | tsa34 | a4 |
| 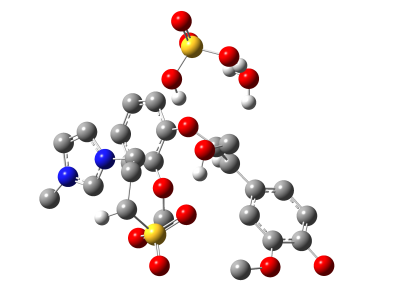 | 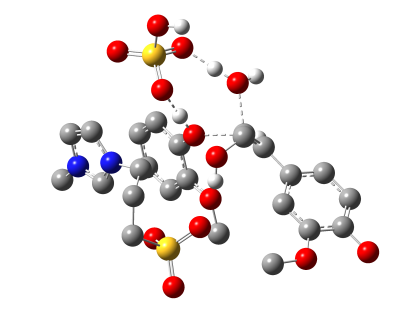 | 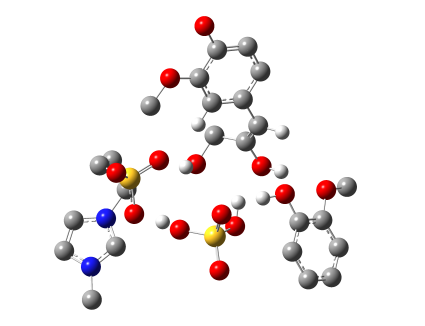 |
| a5 | tsa56 | a6 |

Figure S1 Geometrics of reactants, intermediates, transition states and products in route A. The unimportant hydrogen atoms in the structures are omitted.

| 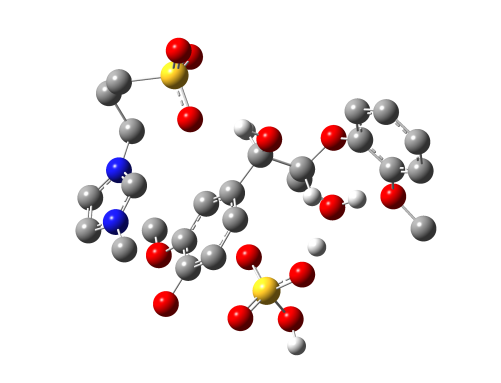 | 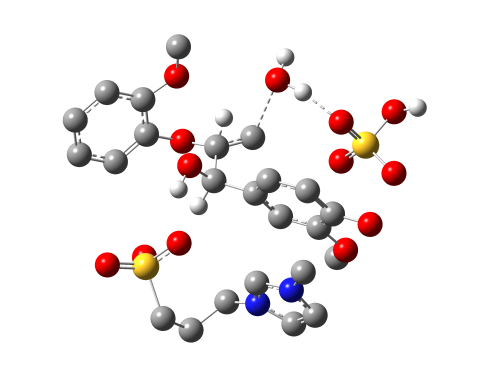 | 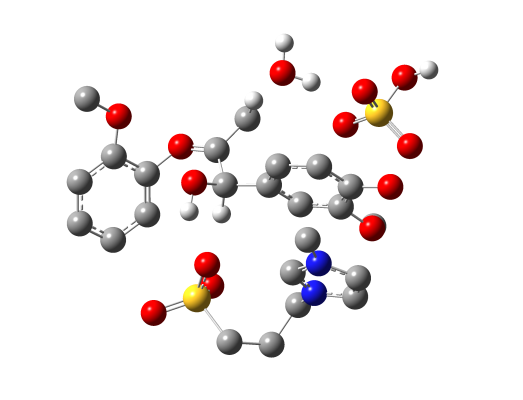 |
| --- | --- | --- |
| b1 | tsb12 | b2 |
| 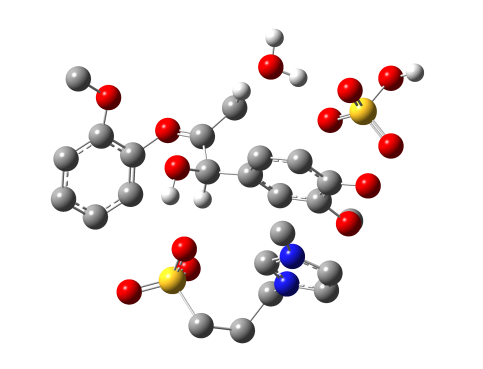 | 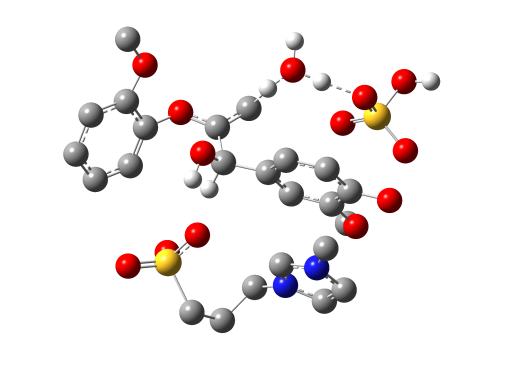 | 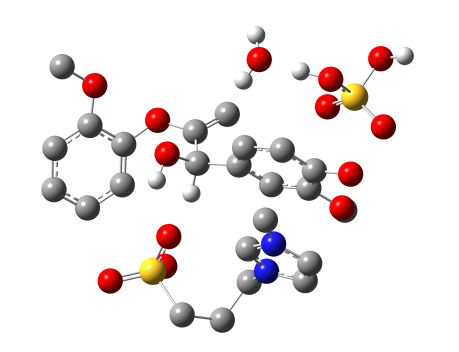 |
| b3 | tsb34 | b4 |
| 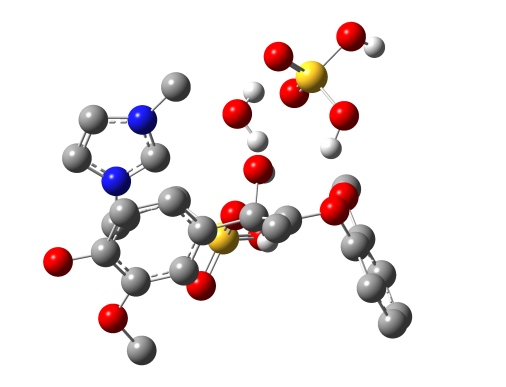 | 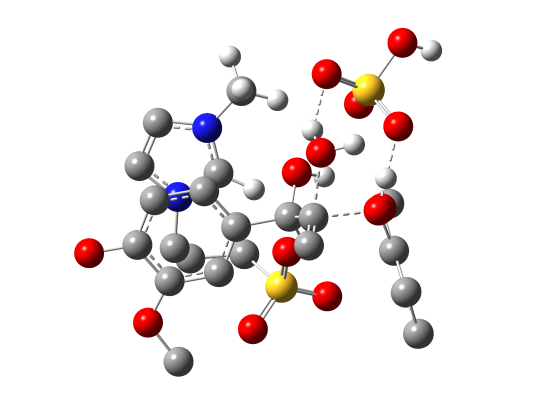 | 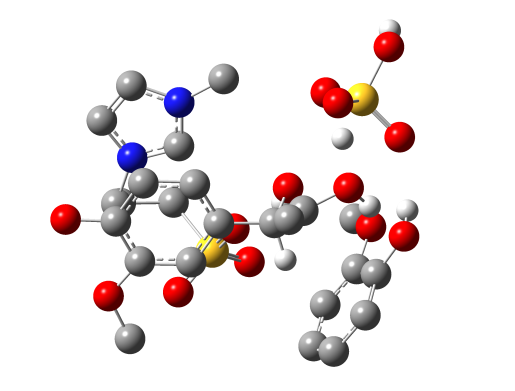 |
| b5 | tsb56 | b6 |

Figure S2 Geometrics of reactants, intermediates, transition states and products in route B. The unimportant hydrogen atoms in the structures are omitted.

| 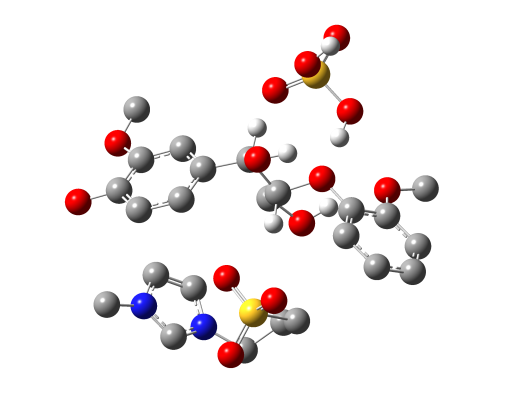 | 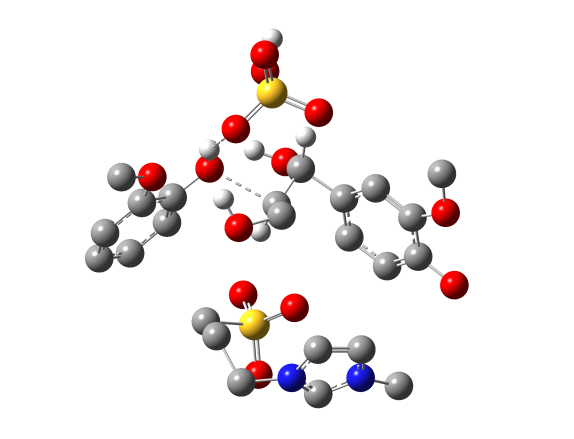 | 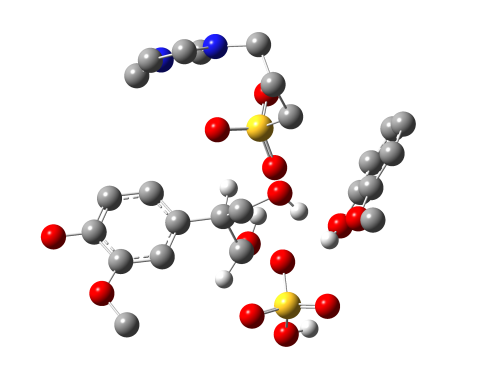 |
| --- | --- | --- |
| c1 | tsc12 | c2 |
| 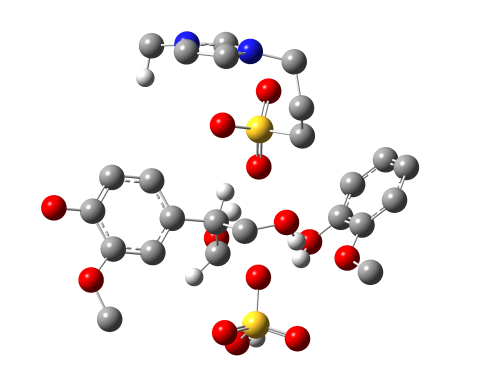 | 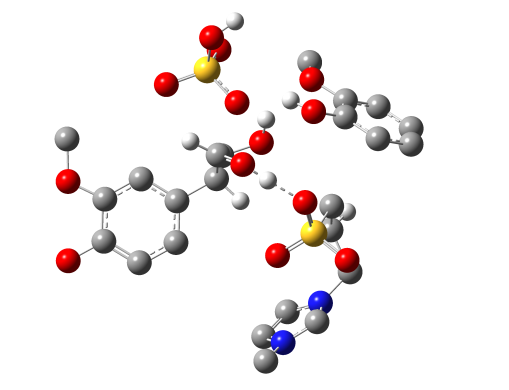 | 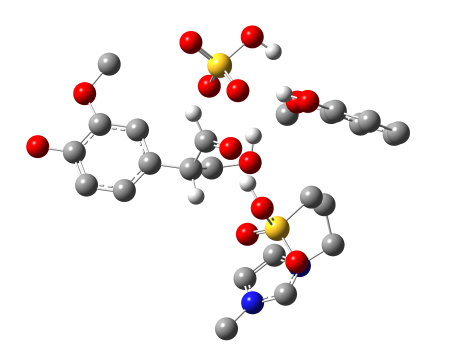 |
| c3 | tsc34 | c4 |

Figure S3 Geometrics of reactants, intermediates, transition states and products in route C. The unimportant hydrogen atoms in the structures are omitted.

Table S1 Electron properties at bond critical points of transition states.

| **Structure** | **Bond** | **Length (Å)** | **BCP** | **ρ** | **▽^2^ρ** | **Sign(λ_2_)ρ** |
| --- | --- | --- | --- | --- | --- | --- |
| ta12 | O42…C17 | 2.327 | 159 | 0.032 | 0.092 | -0.032 |
|  | O42-H75…O71 | 1.815 | 108 | 0.031 | 0.107 | -0.031 |
|  | O35-H36…O37 | 1.895 | 183 | 0.027 | 0.089 | -0.027 |
| ta56 | O21…C19 | 1.921 | 99 | 0.079 | 0.139 | -0.079 |
|  | O21…H75 | 1.047 | 105 | 0.267 | -1.326 | -0.267 |
|  | O71…H75 | 1.465 | 102 | 0.080 | 0.142 | -0.080 |
|  | O72…H20 | 1.460 | 77 | 0.081 | 0.158 | -0.081 |
|  | O42…C19 | 1.967 | 84 | 0.071 | 0.135 | -0.071 |
|  | O42…H20 | 1.028 | 78 | 0.283 | -1.475 | -0.283 |
|  | O35-H36…O62 | 1.831 | 166 | 0.029 | 0.102 | -0.029 |
|  | C46-H50…O63 | 1.898 | 195 | 0.029 | 0.098 | -0.029 |
| tb12 | O42-H43…O63 | 1.80684 | 89 | 0.039 | 0.129 | -0.039 |
|  | C46-H50…O63 | 1.95993 | 100 | 0.026 | 0.087 | -0.026 |
|  | O72…H74 | 1.66396 | 179 | 0.081 | 0.100 | -0.081 |
|  | O35…C32 | 2.29061 | 168 | 0.167 | -0.142 | -0.167 |
| tb56 | O21…C19 | 1.88196 | 184 | 0.088 | 0.119 | -0.088 |
|  | O21…H75 | 1.03292 | 175 | 0.278 | -1.490 | -0.278 |
|  | O71…H75 | 1.50328 | 164 | 0.070 | 0.154 | -0.070 |
|  | O35…C19 | 1.89078 | 157 | 0.085 | 0.129 | -0.085 |
|  | O35-H36…O73 | 1.8834 | 105 | 0.029 | 0.090 | -0.029 |
| tc12 | O21…C19 | 2.22975 | 154 | 0.038 | 0.110 | -0.038 |
|  | O21…H49 | 0.99244 | 160 | 0.321 | -1.982 | -0.321 |
|  | O48…H49 | 1.75741 | 196 | 0.274 | 0.928 | -0.274 |


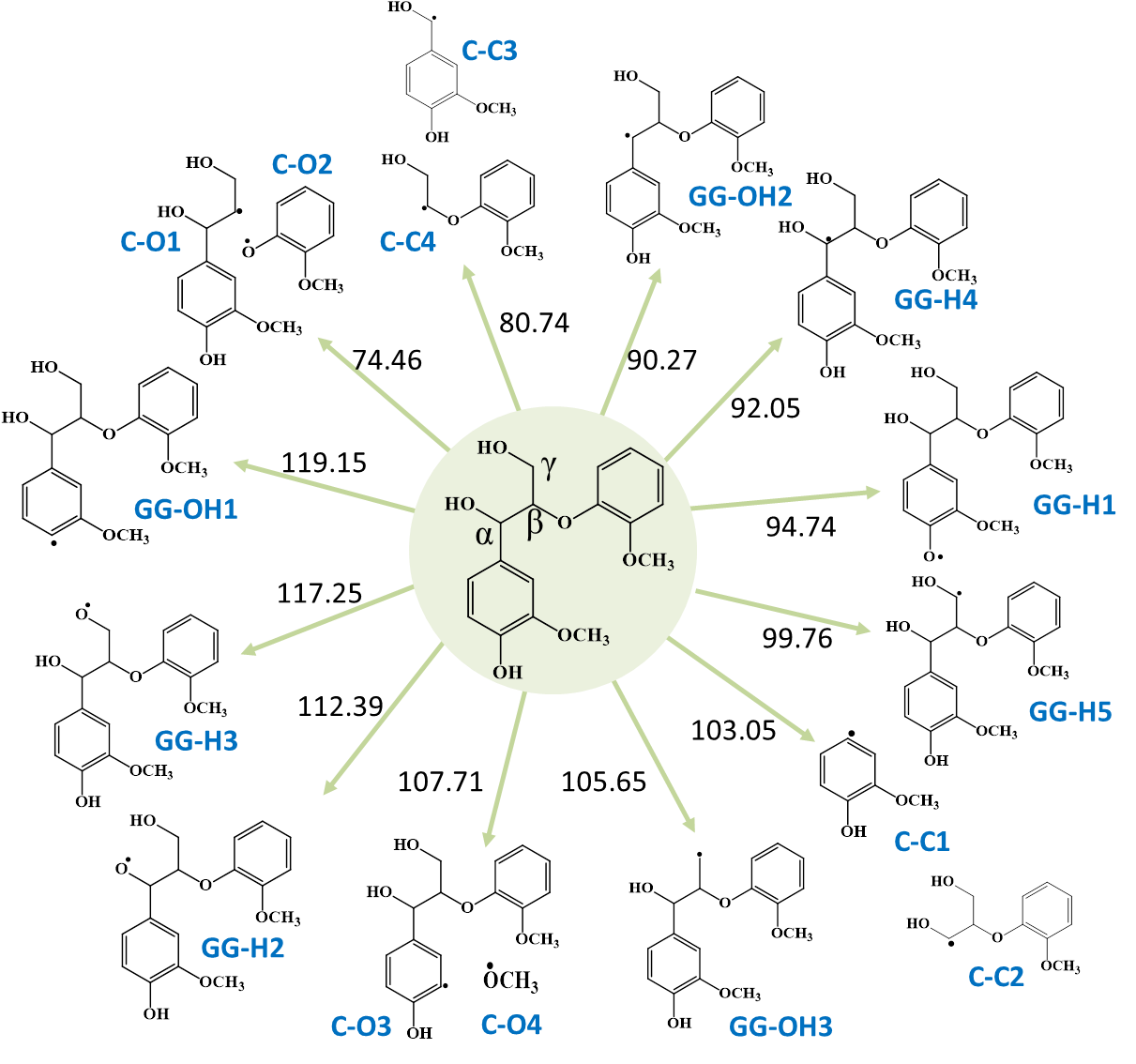


Figure S4 Routes of bond dissociation of lignin model, GG. Bond dissociation energies are summarized in Table S2.

Table S2 Bond dissociation energy of different types of bonds in model GG at M06-2x/6-311+G(d,p) level. Bonds to break are indicated in blue color.

| Bonds to break | Product radicals | | Bond dissociation energy (kcal/mol) |
| --- | --- | --- | --- |
| β-C-O-4 | C-O1 | C-O2 | 74.46 |
| α-C-β-C | C-C3 | C-C4 | 80.74 |
| α-C-OH | GG-OH2 | OH | 90.27 |
| C-H | GG-H4 | H | 92.05 |
| O-H | GG-H1 | H | 94.74 |
| C-H | GG-H5 | H | 99.76 |
| Benzene-α-C | C-C1 | C-C2 | 103.05 |
| γ-C-OH | GG-OH3 | OH | 105.65 |
| Benzene-OCH_3_ | C-O3 | C-O4 | 107.71 |
| O-H | GG-H2 | H | 112.39 |
| Benzene-OCH_3_ | GG-H3 | H | 117.25 |
| Benzene-OH | GG-OH1 | OH | 119.15 |







Figure S5 Atom-atom RDFs of the system GG and [C_3_SO_3_Hmim][HSO_4_] at different temperatures.


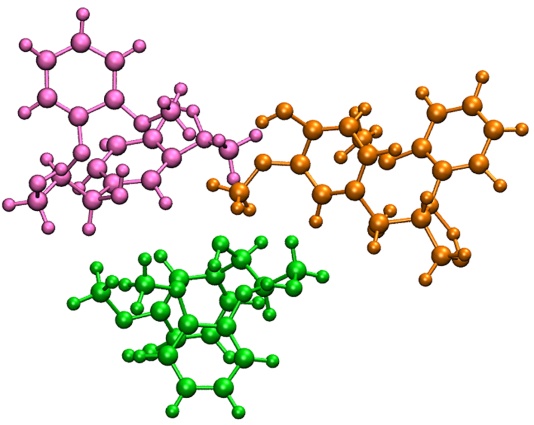

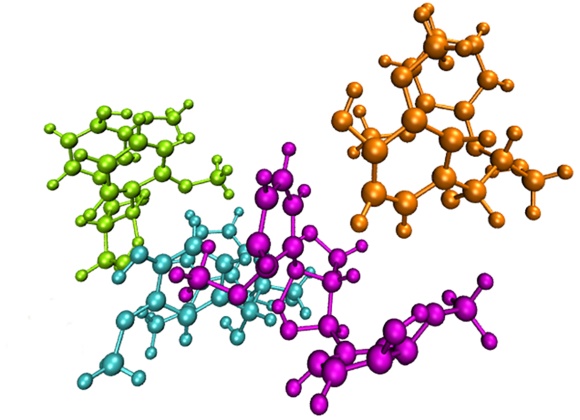


Figure S6 Snapshots of GG clusters in the system of 300K. Left: three GG molecules in a cluster, right: four GG molecules in a cluster. The different molecules are shown in different colors.

Table S3 Rigid scanning to perform the conformational search at the B3LYP/6-31g(d,p) level and the lowest-energy conformation was marked by red circles. The dihedral of the lowest-energy conformation was compared with the original dihedral of the structure which was optimized from IRC path at the M06-2X/6-31+g(d,p) level.

| Scan coordinate | Scanned dihedrals | Original dihedrals |
| --- | --- | --- |
| Route A | | |
| a1: 56-55-52-47 | -174.00 | -173.81 |
| 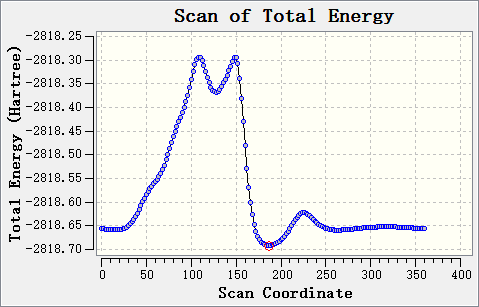 | 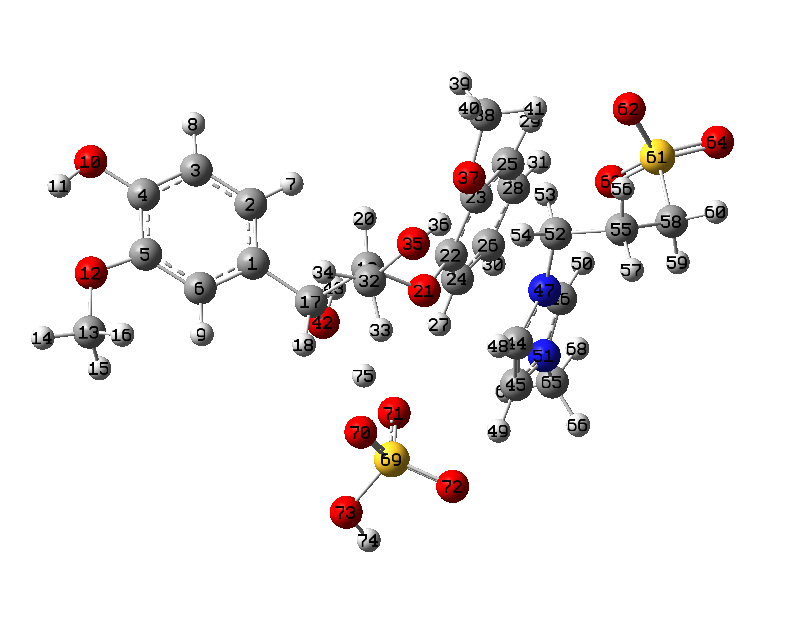 | |
| a2: 56-55-52-47 | 176.00 | -175.43 |
| 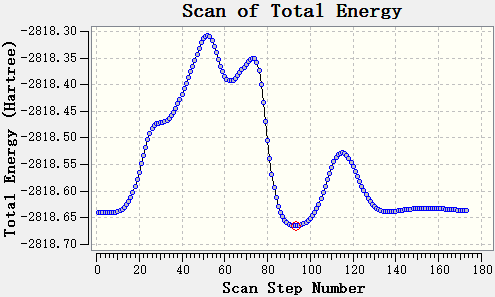 | 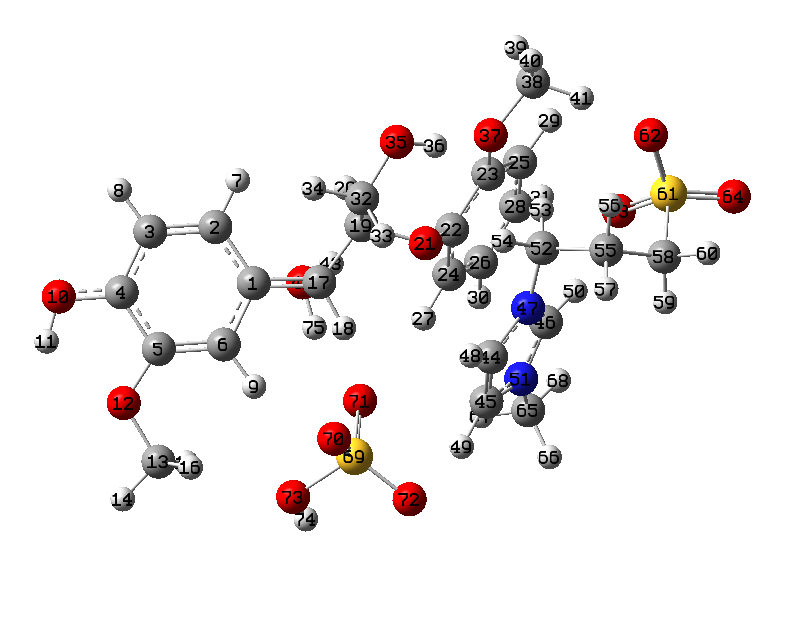 | |
| a3: 56-55-52-47 | -176.00 | -174.56 |
| 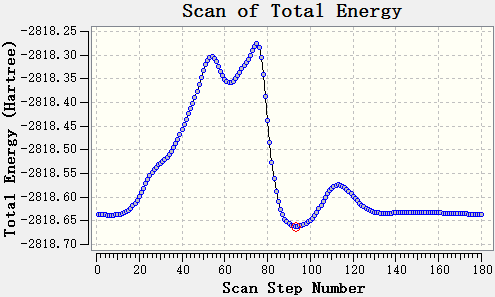 | 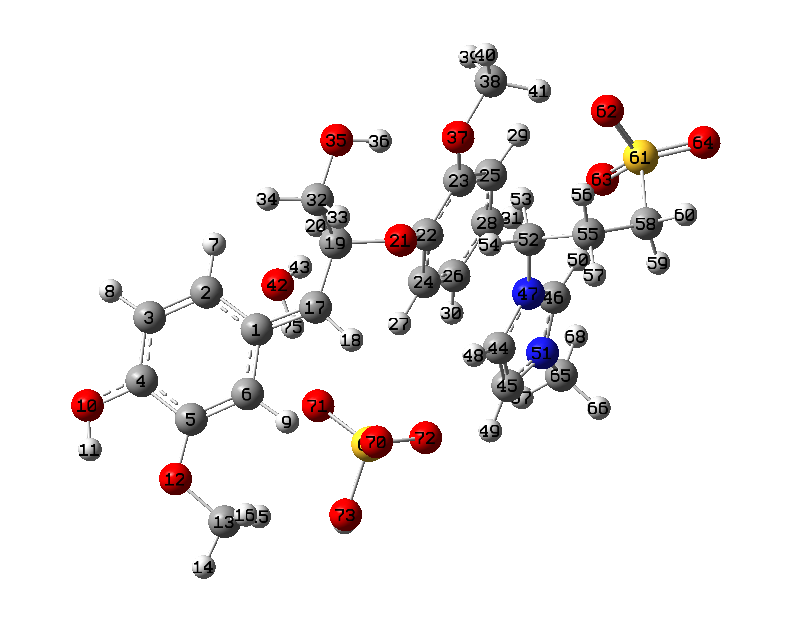 | |
| a4: 56-55-52-47 | -176.00 | -175.28 |
| 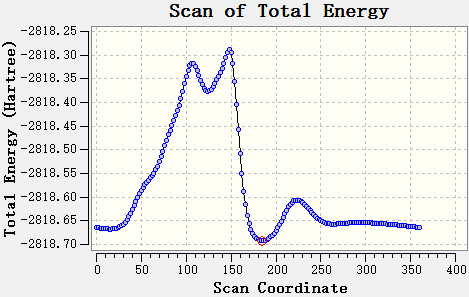 | 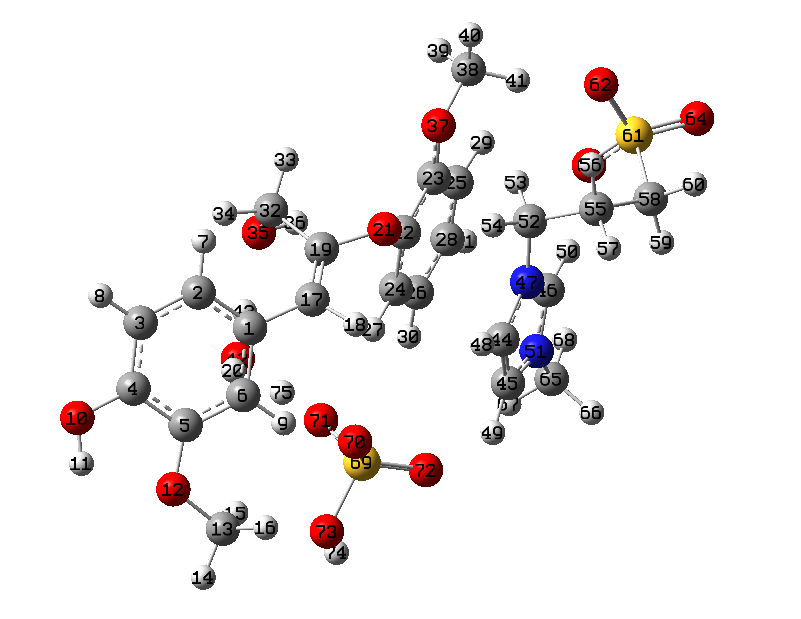 | |
| a5: 11-5-2-1 | -134.00 | -133.51 |
| 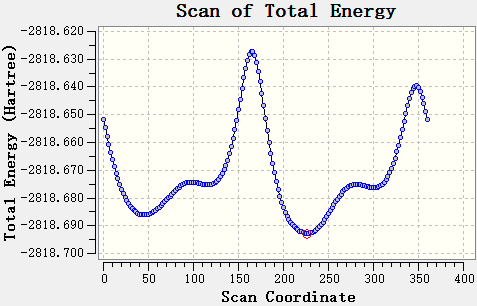 | 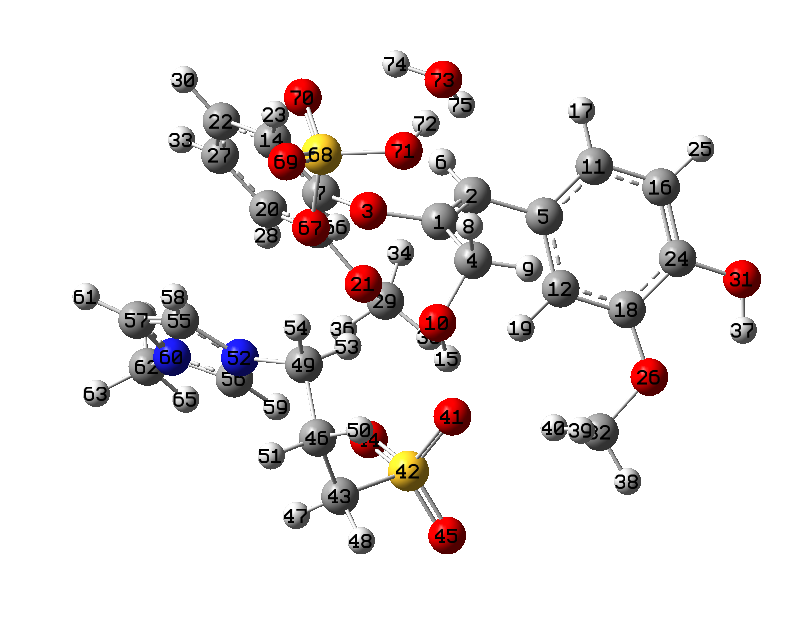 | |
| a6: 32-29-28-27 | 40.00 | 39.27 |
| 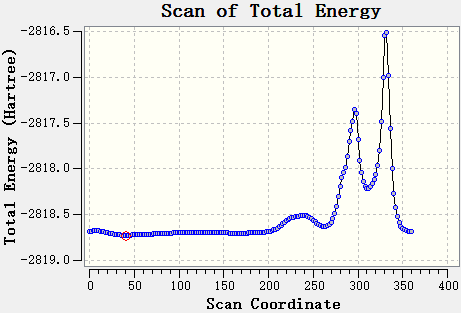 | 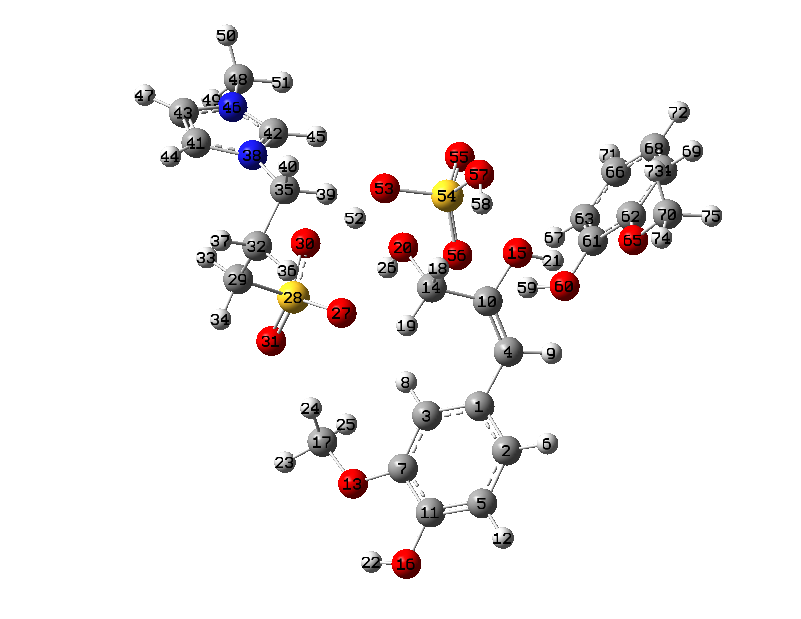 | |
| Route B | | |
| b1: 23-22-21-19 | 116.00 | 95.57 |
| 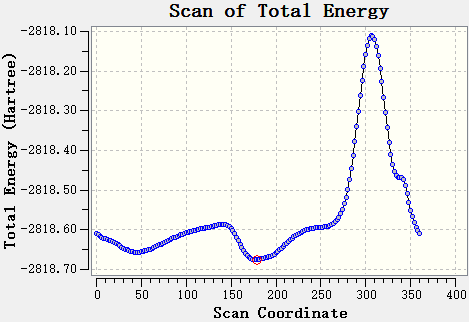 | 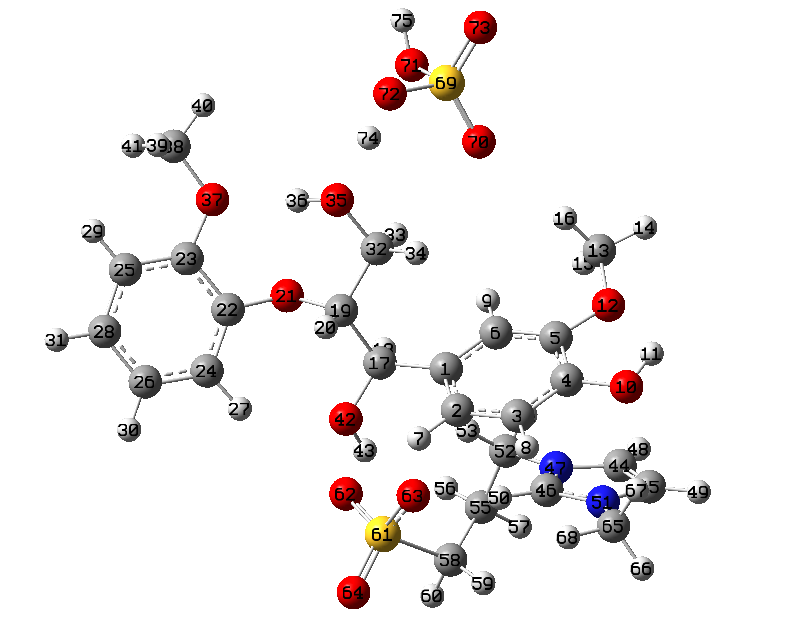 | |
| b2: 56-55-52-47 | -174.00 | -172.53 |
| 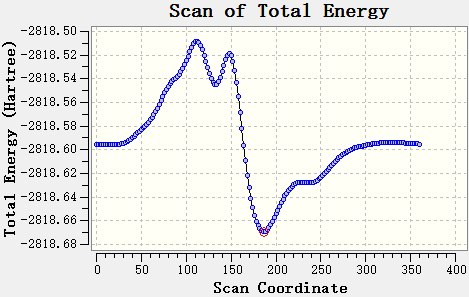 | 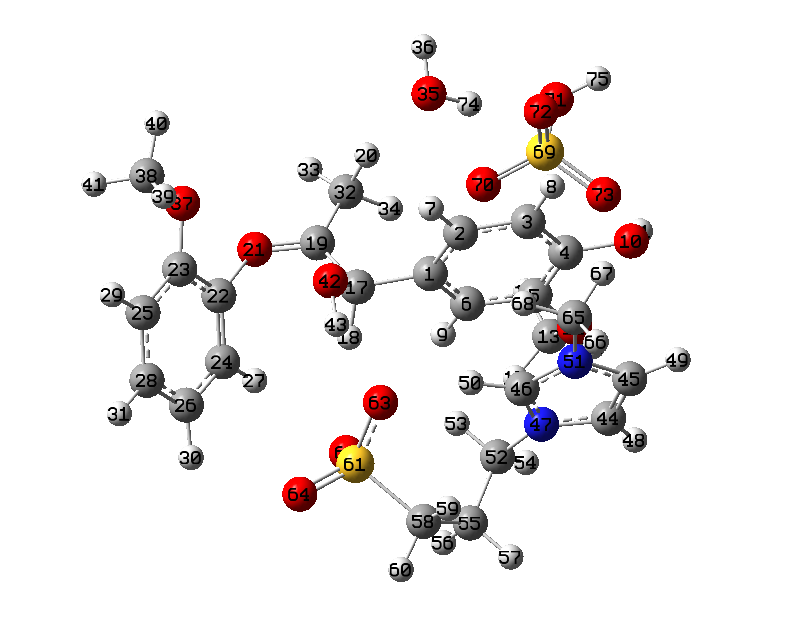 | |
| b3: 56-55-52-47 | -174.00 | -172.53 |
| 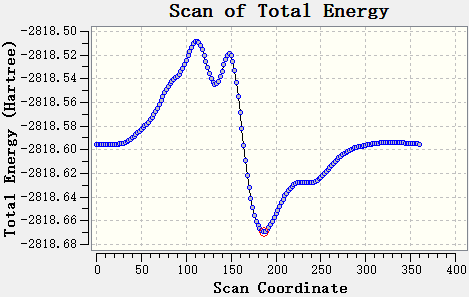 | 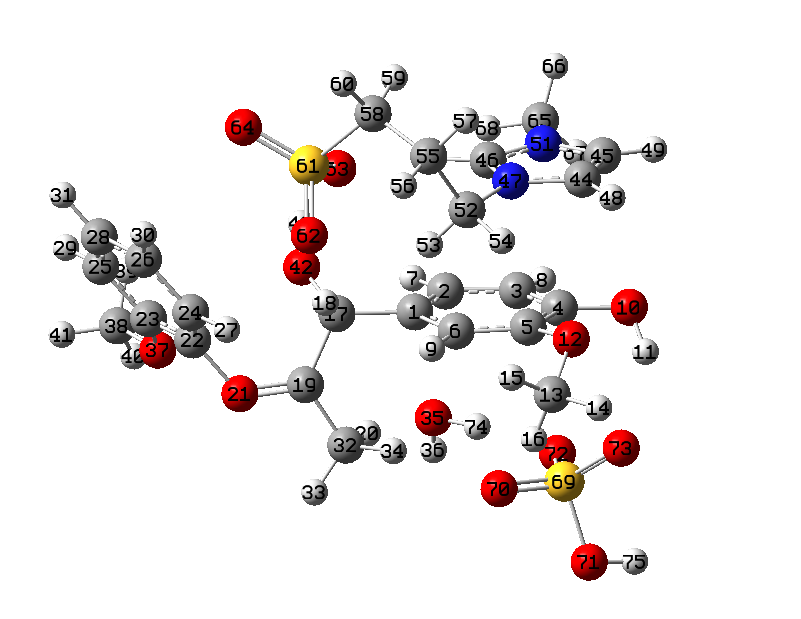 | |
| b4: 22-21-19-17 | -28.00 | -21.87 |
| 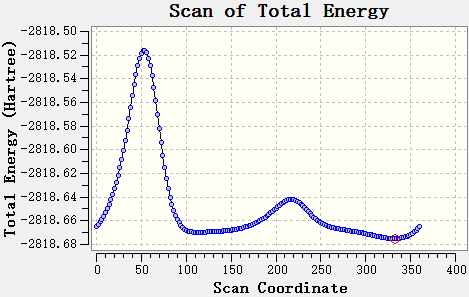 | 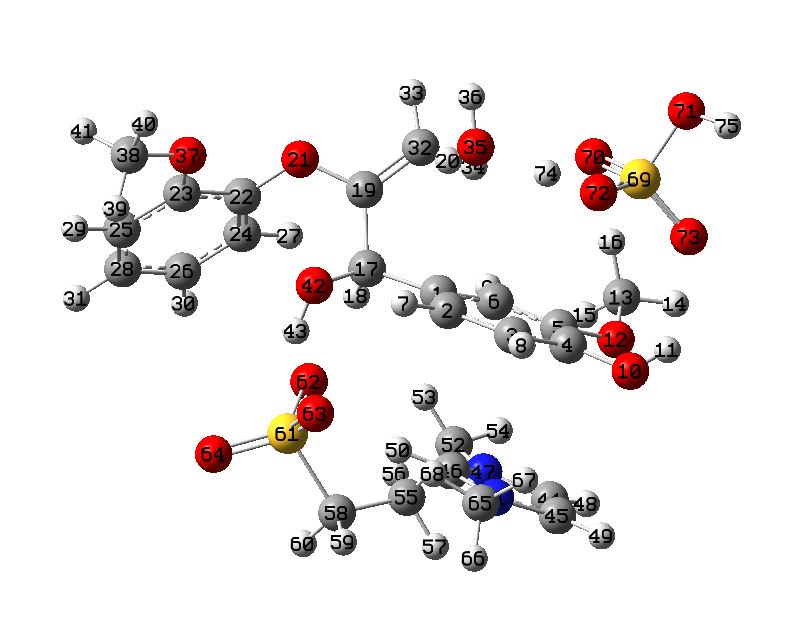 | |
| b5: 56-55-52-47 | -172.00 | -171.67 |
| 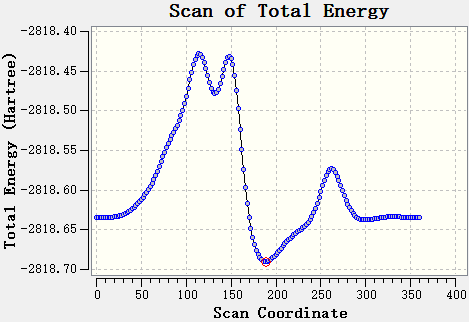 | 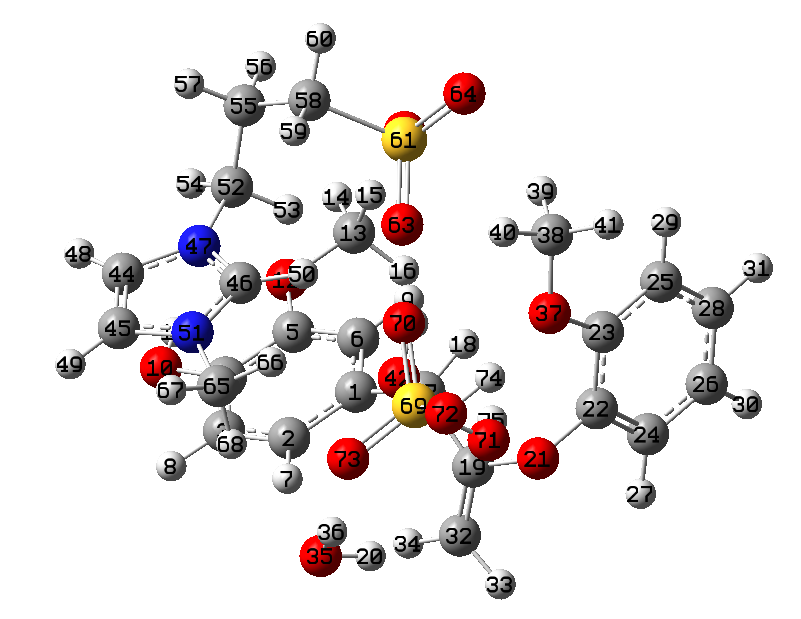 | |
| b6: 56-55-52-47 | -172.00 | -171.72 |
| 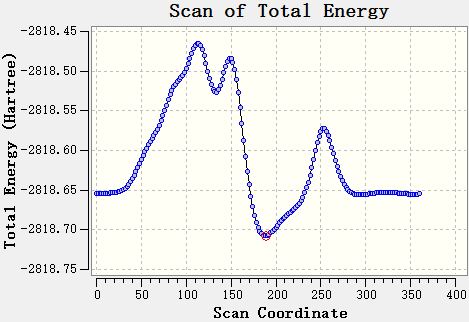 | 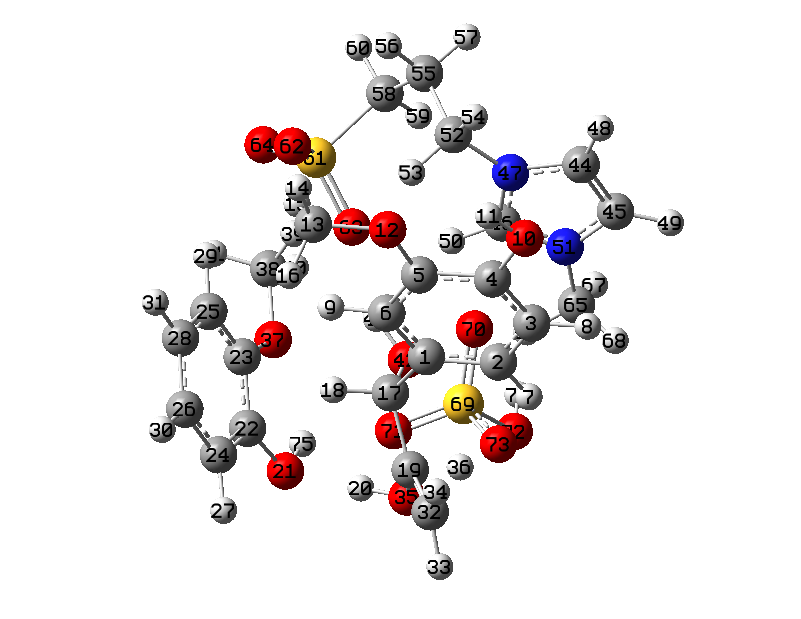 | |
| Route C: | | |
| c1: 66-65-62-59 | -164.00 | -165.84 |
| 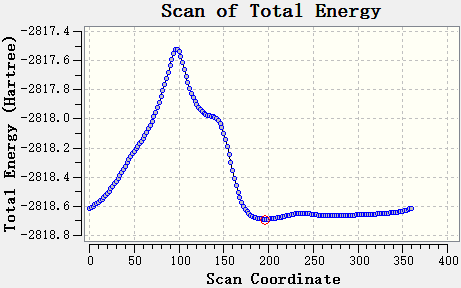 | 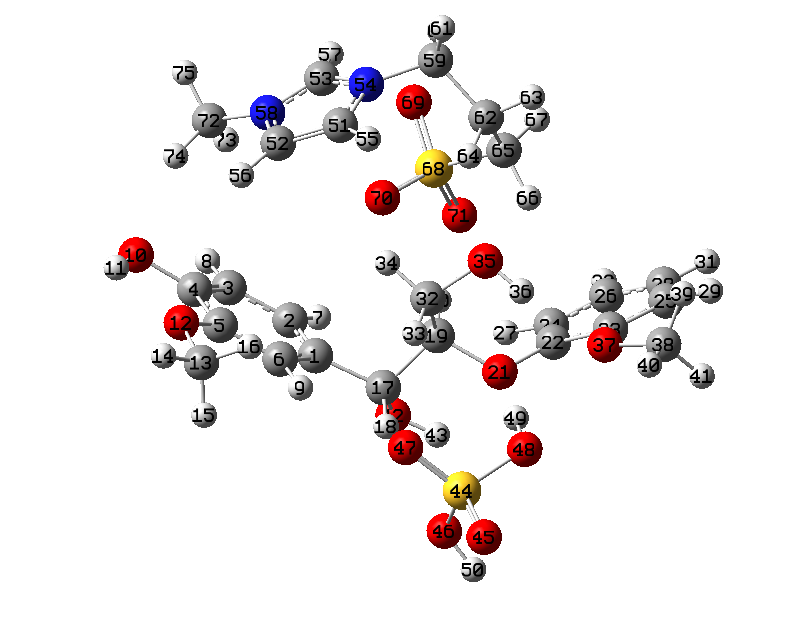 | |
| c2: 42-39-36-35 | -52.00 | -49.85 |
| 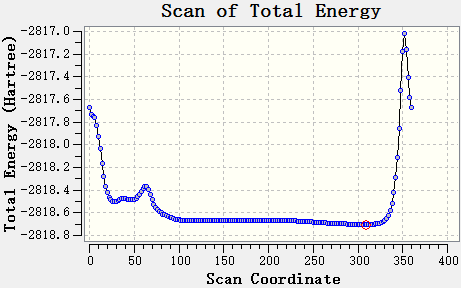 | 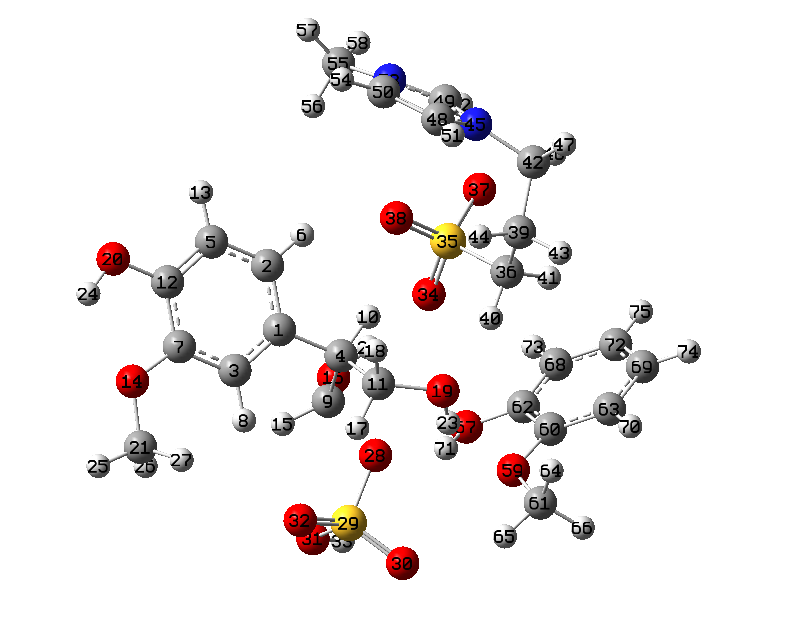 | |
| c3: 42-39-36-35 | -52.00 | -49.85 |
| 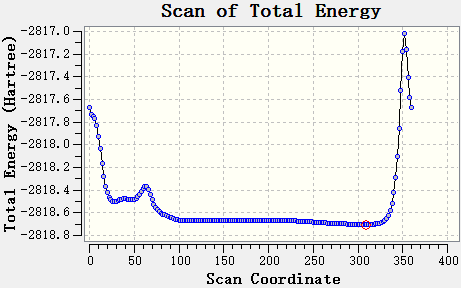 | 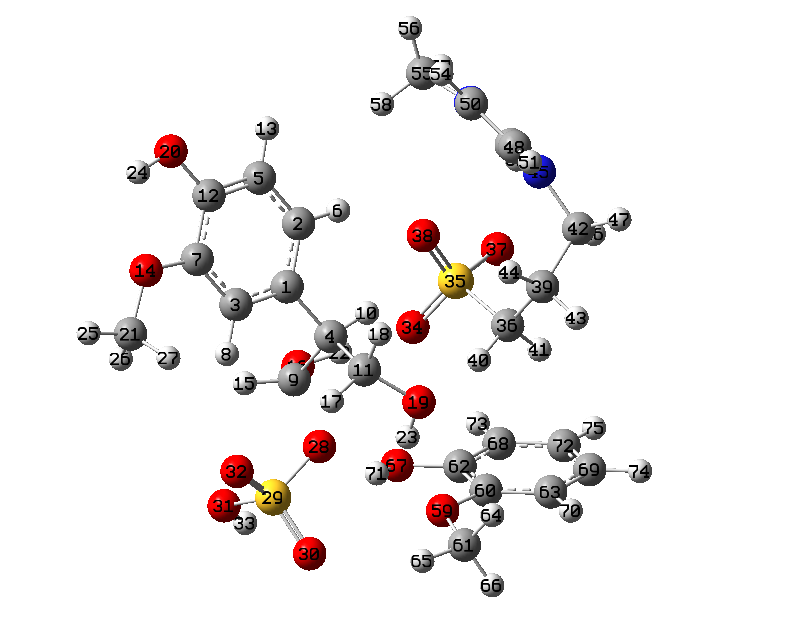 | |
| c4: 39-36-33-30 | 86.00 | 82.92 |
| 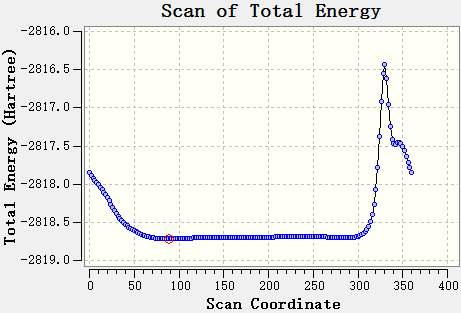 | 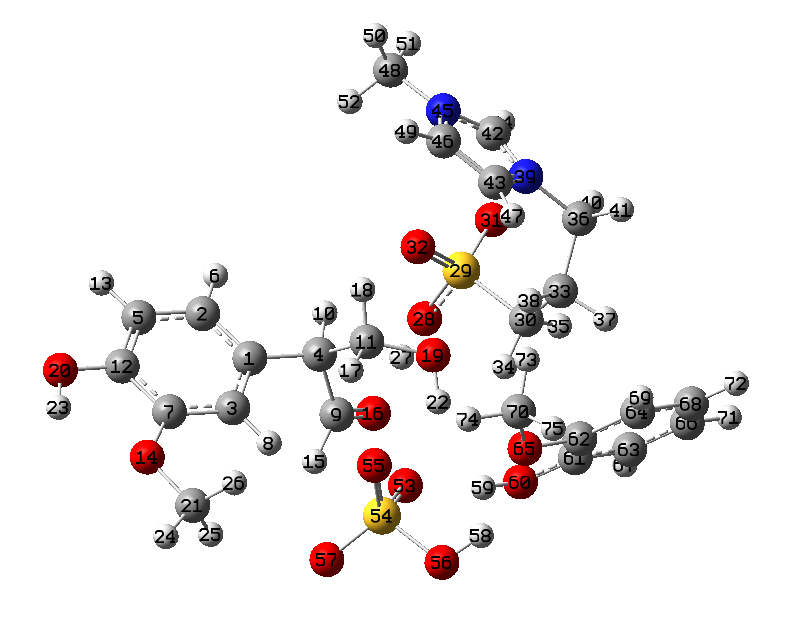 | |

| 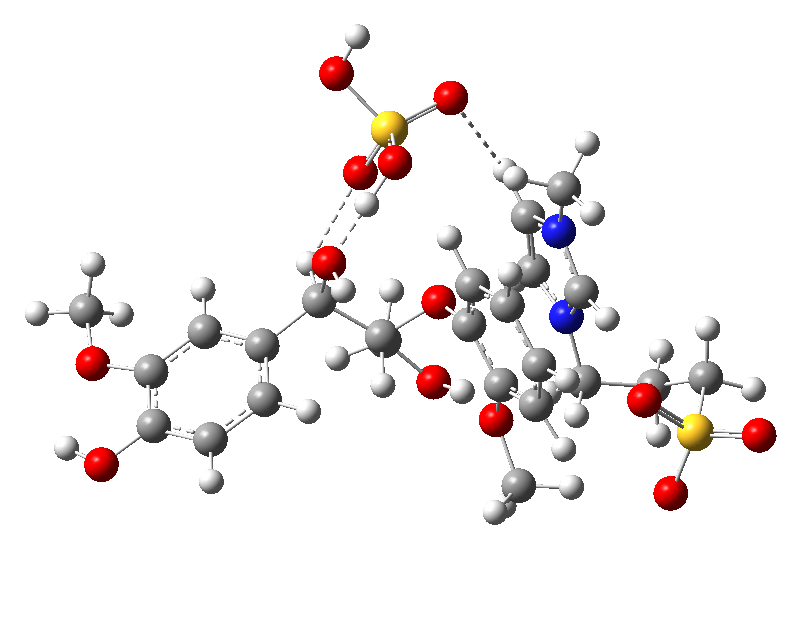 | 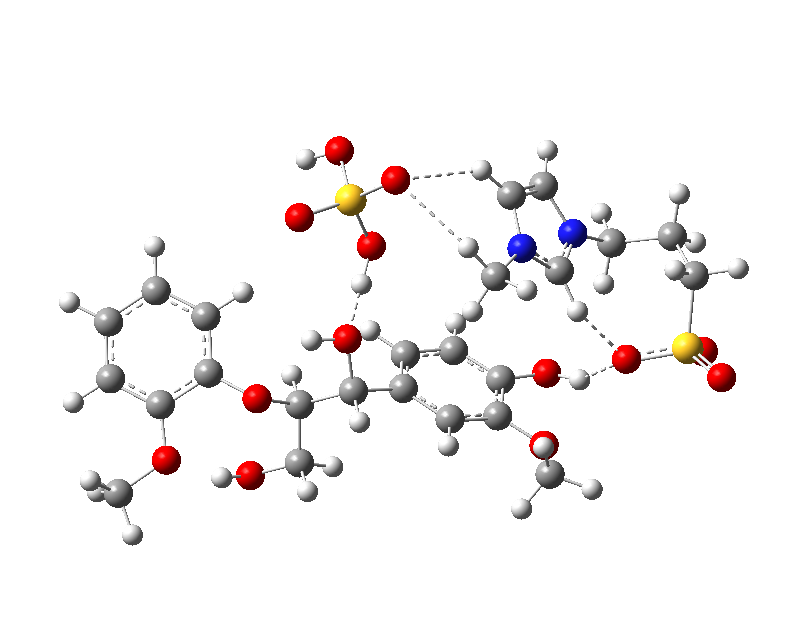 | 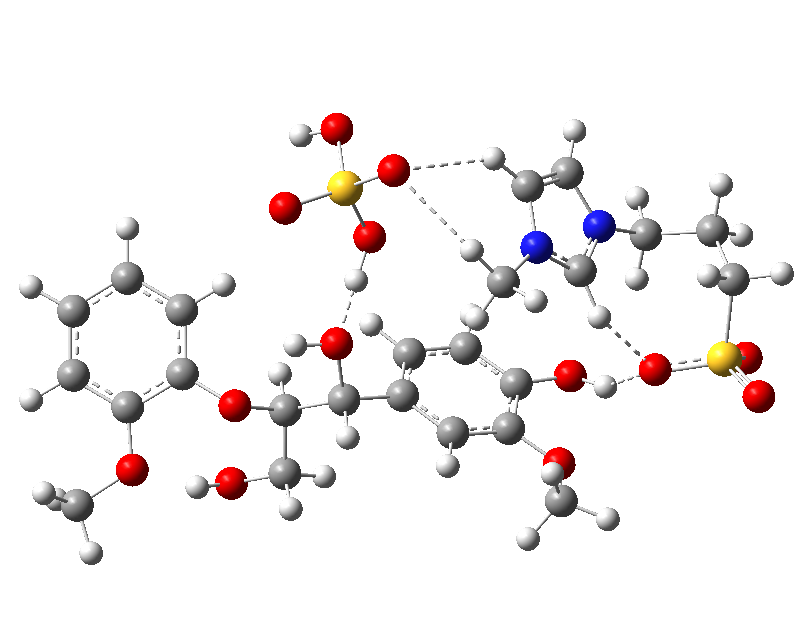 |
| --- | --- | --- |
| a1 (-52.82 kcal/mol) | a1-2 (-41.15 kcal/mol) | a1-3 (-39.36 kcal/mol) |
| 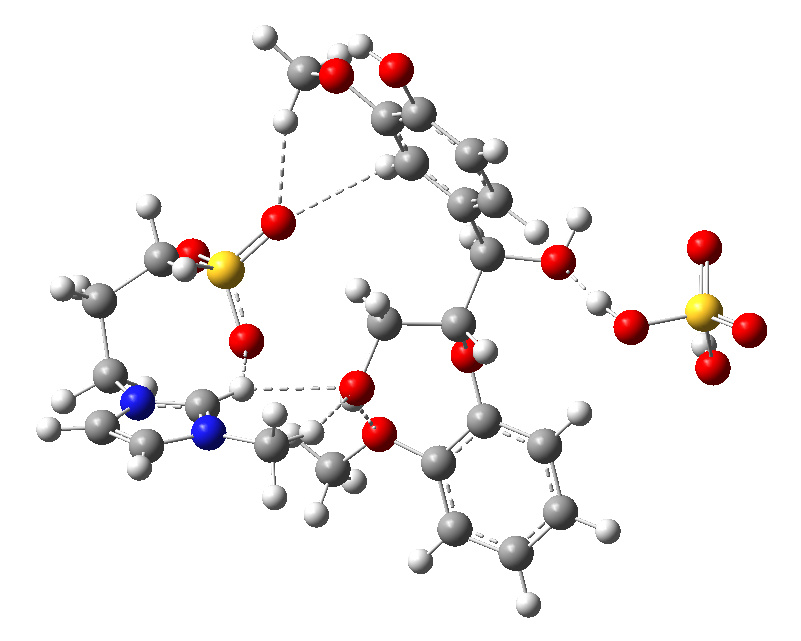 | 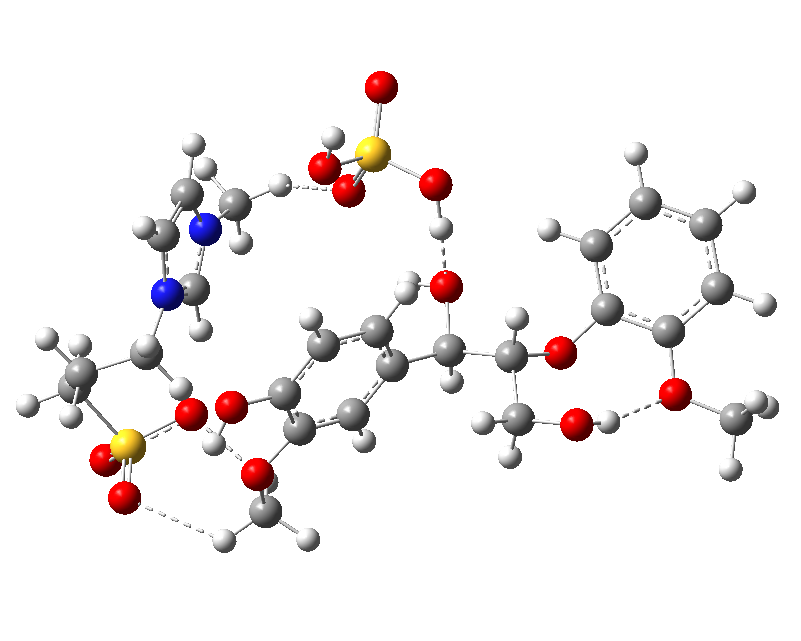 | 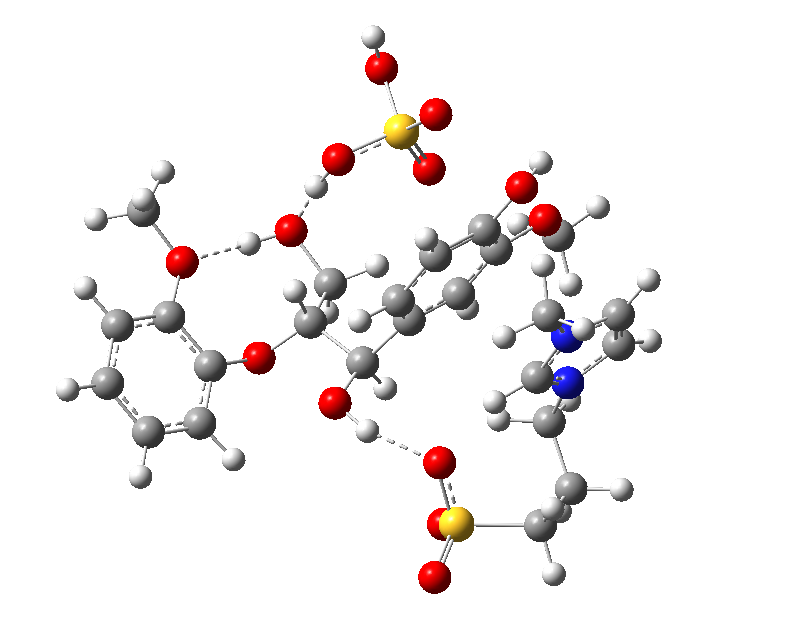 |
| a1-4 (-39.26 kcal/mol) | a1-5 (-30.16 kcal/mol) | b1 (-44.10 kcal/mol) |
| 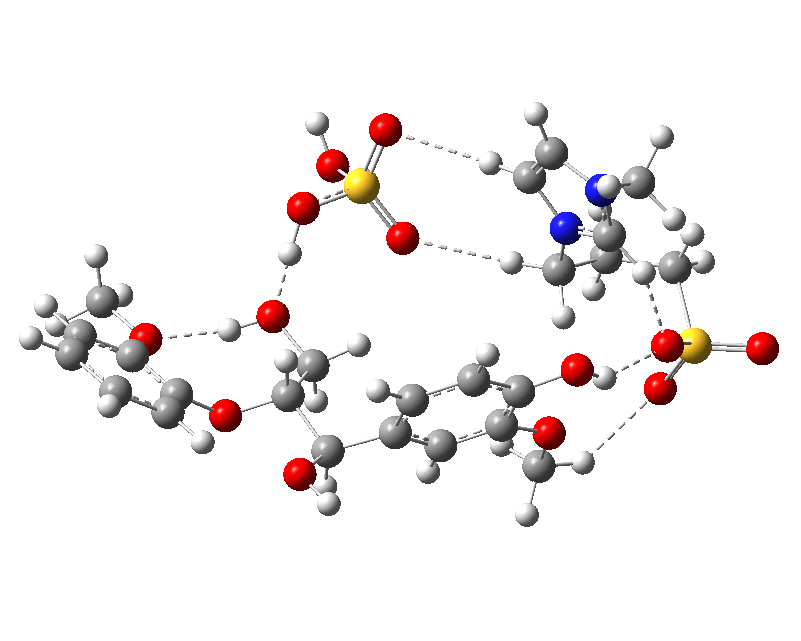 | 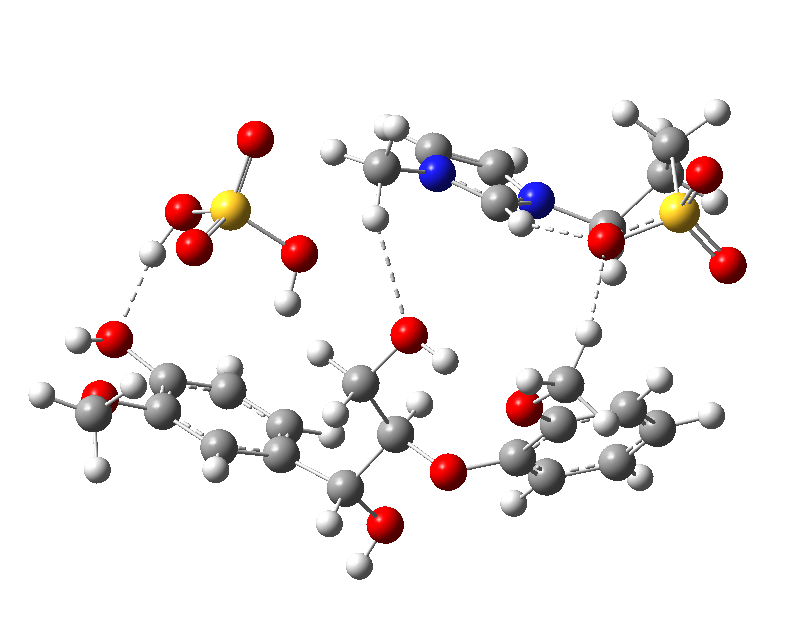 | 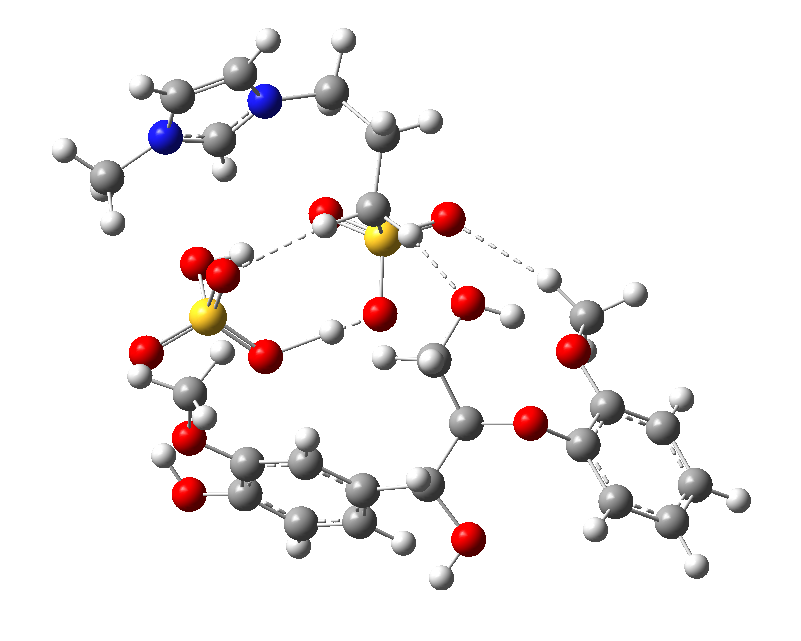 |
| b1-1 (-42.53 kcal/mol) | b1-2 (-35.95 kcal/mol) | b1-3 (-14.36 kcal/mol) |
| 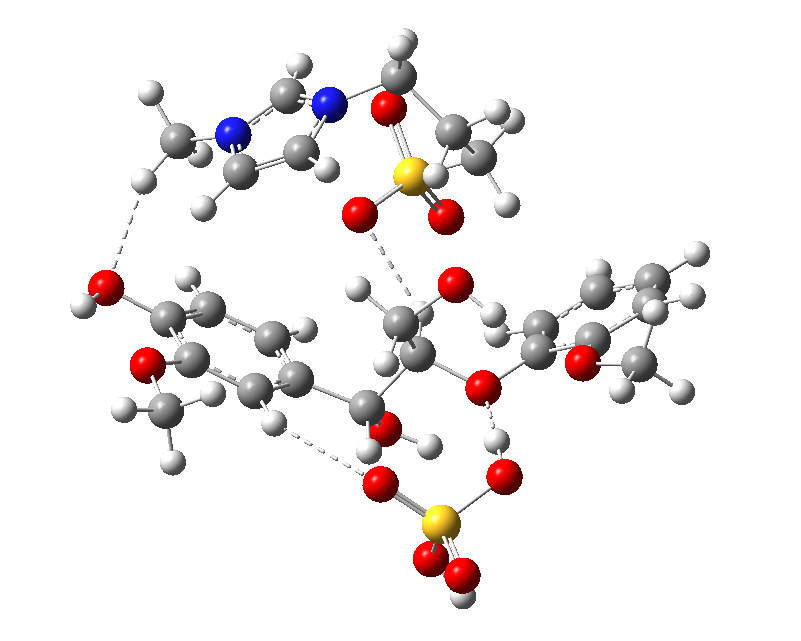 | 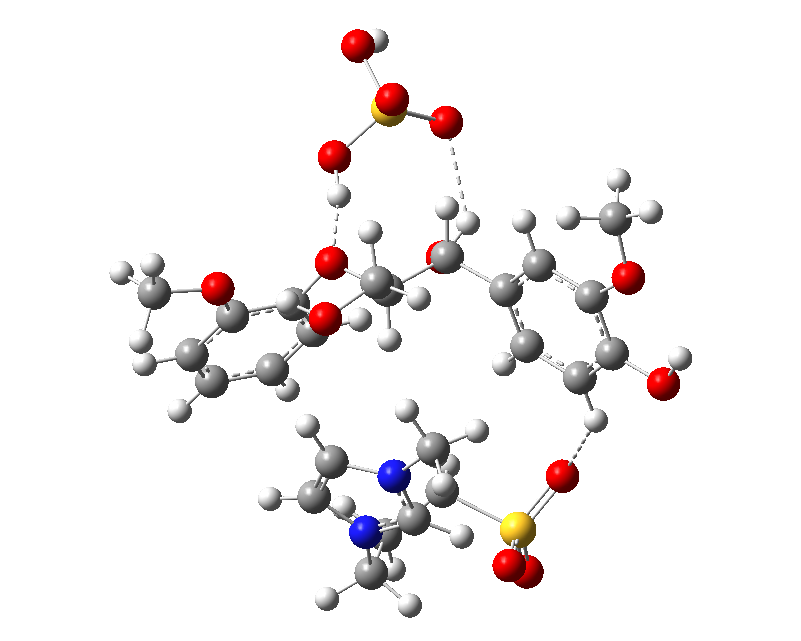 | 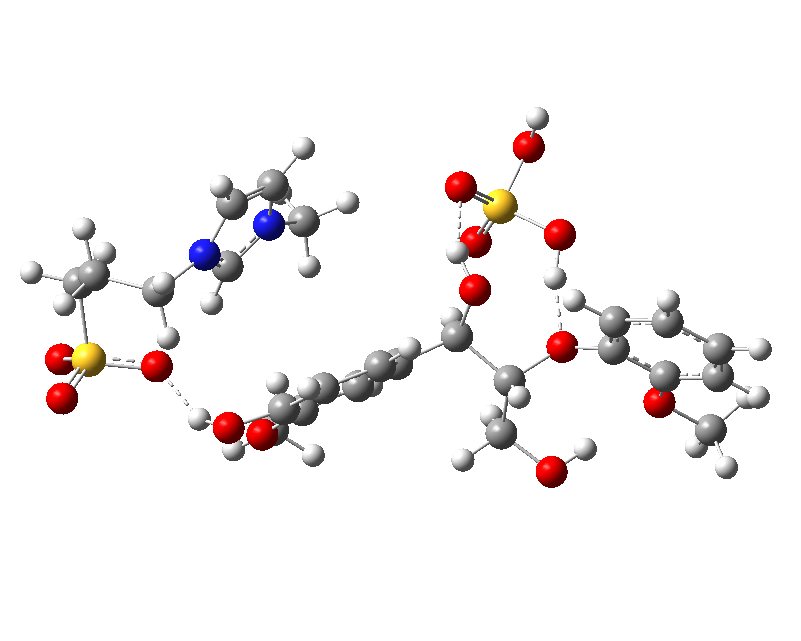 |
| c1 (-37.16 kcal/mol) | c1-2 (36.19 kcal/mol) | c1-3 (-35.97 kcal/mol) |

Figure S7 Stable geometries of the interaction between GG and zwitterion/H_2_SO_4_ which were optimized at the B3LYP/6-31g(d,p) level and interaction energies were refined at the M06-2X/6-311+g(d,p) level with counterpoise method.
